# Author Correction: Long noncoding RNA Malat1 protects against osteoporosis and bone metastasis

**DOI:** 10.1038/s41467-024-49356-0

**Published:** 2024-06-10

**Authors:** Yang Zhao, Jingyuan Ning, Hongqi Teng, Yalan Deng, Marisela Sheldon, Lei Shi, Consuelo Martinez, Jie Zhang, Annie Tian, Yutong Sun, Shinichi Nakagawa, Fan Yao, Hai Wang, Li Ma

**Affiliations:** 1 Department of Experimental Radiation Oncology, The University of Texas MD Anderson Cancer Center, Houston, TX 77030 USA; 2 Institute of Basic Medical Sciences, Peking Union Medical College, Chinese Academy of Medical Sciences, Beijing, 100010 China; 3 Department of Endocrine Neoplasia and Hormonal Disorders, The University of Texas MD Anderson Cancer Center, Houston, TX 77030 USA; 4 Department of Kinesiology, Rice University, Houston, TX 77005 USA; 5 Department of Molecular and Cellular Oncology, The University of Texas MD Anderson Cancer Center, Houston, TX 77030 USA; 6 RNA Biology Laboratory, Faculty of Pharmaceutical Sciences, Hokkaido University, Sapporo, 060-0812 Japan; 7 Department of Molecular and Cellular Biology, Roswell Park Comprehensive Cancer Center, Buffalo, NY 14263 USA; 8 The University of Texas MD Anderson Cancer Center UTHealth Houston Graduate School of Biomedical Sciences, Houston, TX 77030 USA; 9 Present Address: Hubei Hongshan Laboratory, College of Biomedicine and Health, Huazhong Agricultural University, Wuhan, Hubei 430070 China

**Keywords:** Mechanisms of disease, Long non-coding RNAs, Metastasis

Correction to: *Nature Communications* 10.1038/s41467-024-46602-3, published online 16 March 2024

The original version of this Article contained an error in Fig. 2b, in which the micro-CT image used as the representative image for male *Malat1-/-* in Fig. 2a was duplicated and presented as the representative image for the female *Malat1+/+* mice.

This error has been now corrected in both the PDF and HTML versions of the Article.

All micro-CT images used to generate the plots in Fig. 2c, d are now provided through the following link:

https://figshare.com/articles/figure/micro-CT_images_for_Figs_2c_and_2d_tif/25683639

Representative images used in Fig. 2a (males): 6297L, 5958L, and 6862R.

Representative images used in Fig. 2b (females): 97R, 79R, and 70R.

