# Supplementary material for: Long noncoding RNA Malat1 protects against osteoporosis and bone metastasis
Source: Nat Commun. 2024 Mar 16;15:2384. doi: 10.1038/s41467-024-46602-3 (PMC10944492; doi:10.1038/s41467-024-46602-3)
Supplement: Supplementary file 1 — Supplementary Information [file 41467_2024_46602_MOESM1_ESM.pdf]

Supplementary Information

Long noncoding RNA Malat1 protects against osteoporosis and bone metastasis

Yang Zhao *et al.*

*Correspondence: Li Ma (lma4@mdanderson.org)

This PDF file includes:

Supplementary Figures 1-11

Other Supplementary Materials for this manuscript include the following:

Supplementary Data 1-5

Source Data

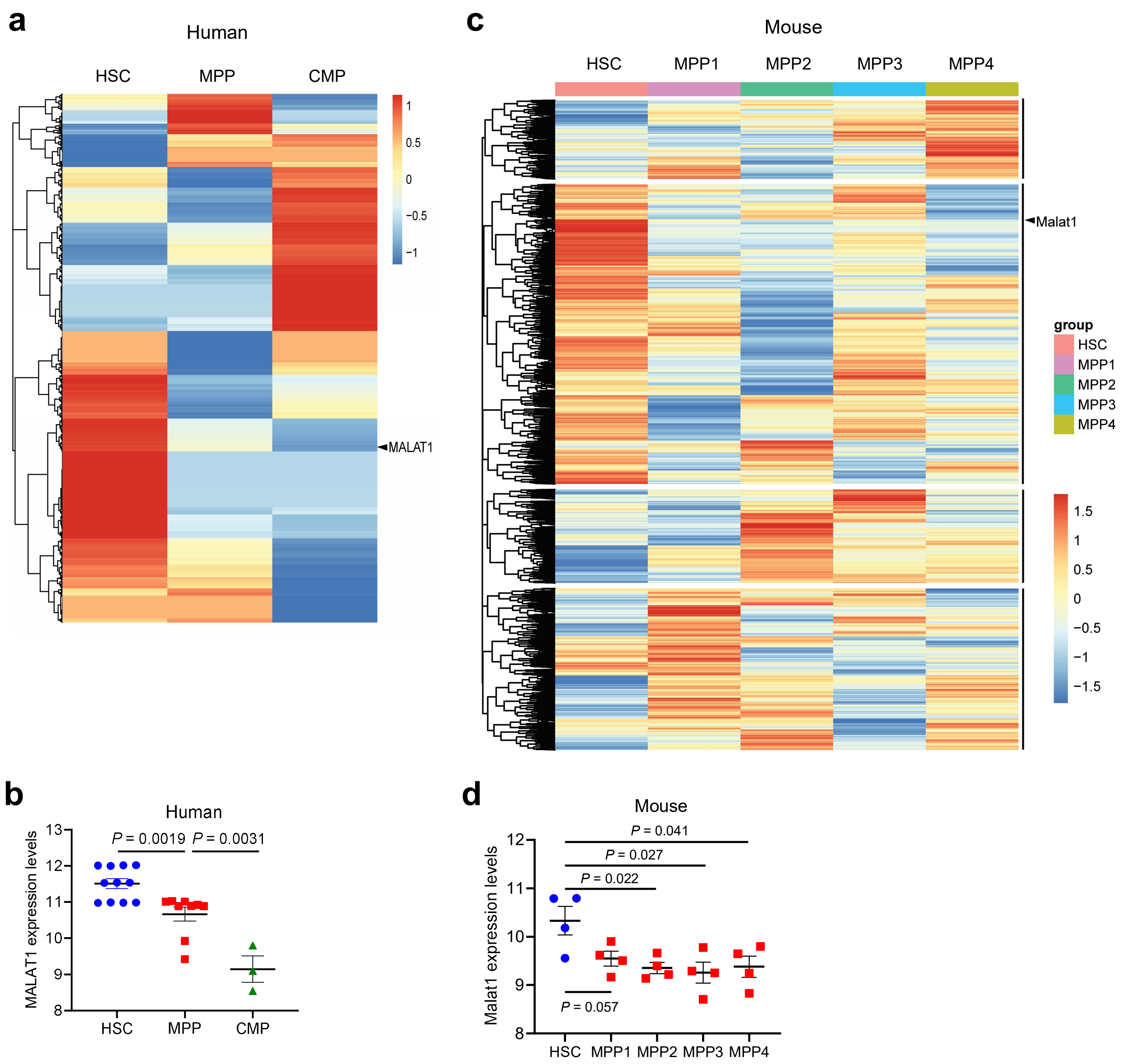

Supplementary Figure 1. Malat1 is downregulated in hematopoietic progenitor cells compared with hematopoietic stem cells in humans and mice.

a. Heatmap of differentially expressed genes among human hematopoietic stem cells (HSC), multipotent progenitors (MPP), and common myeloid progenitors (CMP). Data source: Expression Atlas dataset E-MTAB-3819.

b. Expression levels of MALAT1, calculated as $\log_2$(FPKM +1) values, were quantitated from the RNA-seq results in **a**. n = 11 (from 4 healthy individuals with 1-4 replicates), 9 (from 4 healthy individuals from 1-4 replicates), and 3 (from 3 healthy individuals) samples for HSC, MPP, and CMP groups, respectively.

c. Heatmap of differentially expressed genes among mouse hematopoietic stem cells (HSC) and four groups of multipotent progenitors (MPP). Data source: Expression Atlas dataset E-MTAB-7391.

d. Expression levels of Malat1, calculated as $\log_2$(FPKM +1) values, were quantitated from the RNA-seq results in **c**. n = 4 samples per group, each containing cells pooled from 3 mice.

Statistical significance in **b** and **d** was determined by a two-tailed unpaired t-test. Error bars are s.e.m. Source data are provided as a Source Data file.

Supplementary Figure 2

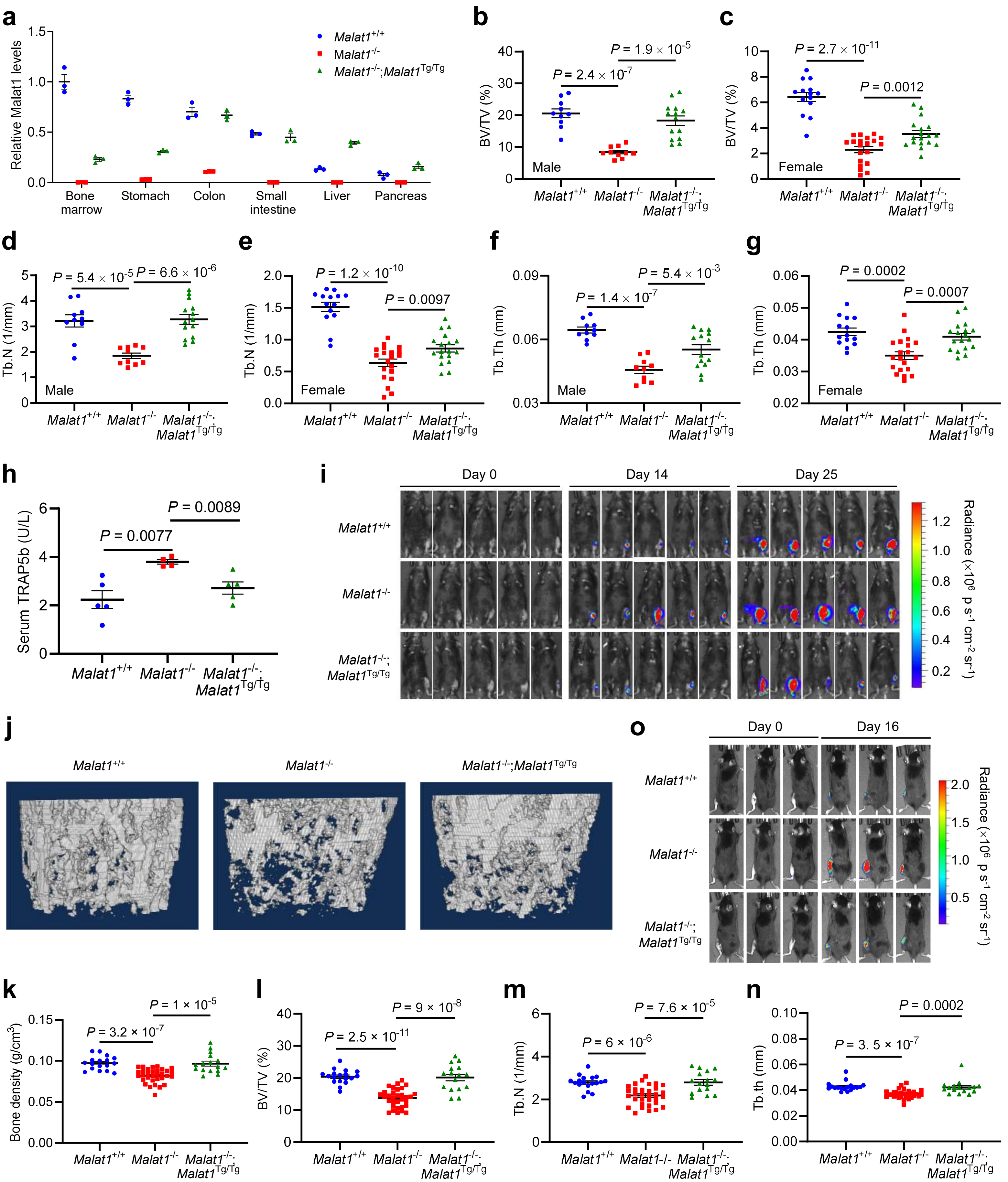

Supplementary Figure 2. Malat1 is a suppressor of osteoporosis and bone metastasis.

a. qPCR of Malat1 in various organs of *Malat1*$^{+/+}$, *Malat1*$^{-/-}$, and *Malat1*$^{-/-}$;*Malat1*$^{Tg/Tg}$ mice. $n = 3$ biological replicates per group.

b-g. μCT-based measurements of the trabecular bone volume per tissue volume (BV/TV, **b** and **c**), trabecular number (Tb.N, **d** and **e**), and trabecular thickness (Tb.th, **f** and **g**) in the femurs from 6-month-old male (n = 5, 5, and 7 mice per group) and female (n = 7, 10, and 9 mice per group) *Malat1*$^{+/+}$, *Malat1*$^{-/-}$, and *Malat1*$^{-/-}$;*Malat1*$^{Tg/Tg}$ mice, with left and right femurs for each mouse measured.

h. ELISA of serum TRAP5b levels in 6-month-old male *Malat1*$^{+/+}$, *Malat1*$^{-/-}$, and *Malat1*$^{-/-}$;*Malat1*$^{Tg/Tg}$ mice. n = 5, 4, and 5 mice per group.

i. 6-month-old *Malat1*$^{+/+}$, *Malat1*$^{-/-}$, and *Malat1*$^{-/-}$;*Malat1*$^{Tg/Tg}$ mice received intratibial injection of 5,000 B16F1 melanoma cells. Bioluminescent imaging of live animals was performed at the indicated times.

j. Representative μCT images of 3D bone structures of the femurs from 3-month-old female *Malat1*$^{+/+}$, *Malat1*$^{-/-}$, and *Malat1*$^{-/-}$;*Malat1*$^{Tg/Tg}$ mice.

k-n. μCT-based measurements of the bone mineral density (BMD, **k**), trabecular bone volume per tissue volume (BV/TV, **l**), trabecular number (Tb.N, **m**), and trabecular thickness (Tb.th, **n**) in the femurs from 3-month-old female *Malat1*$^{+/+}$, *Malat1*$^{-/-}$, and *Malat1*$^{-/-}$;*Malat1*$^{Tg/Tg}$ mice, with left and right femurs for each mouse measured. n = 9, 16, and 8 mice per group.

o. 3-month-old female *Malat1*$^{+/+}$, *Malat1*$^{-/-}$, and *Malat1*$^{-/-}$;*Malat1*$^{Tg/Tg}$ mice received intratibial injection of 2×10^5 EO771 mammary tumor cells. Bioluminescent imaging of live animals was performed at day 0 and day 16.

Statistical significance in **b**-**h** and **k-n** was determined by a two-tailed unpaired *t*-test. Error bars are s.e.m. Source data are provided as a Source Data file.

Supplementary Figure 3

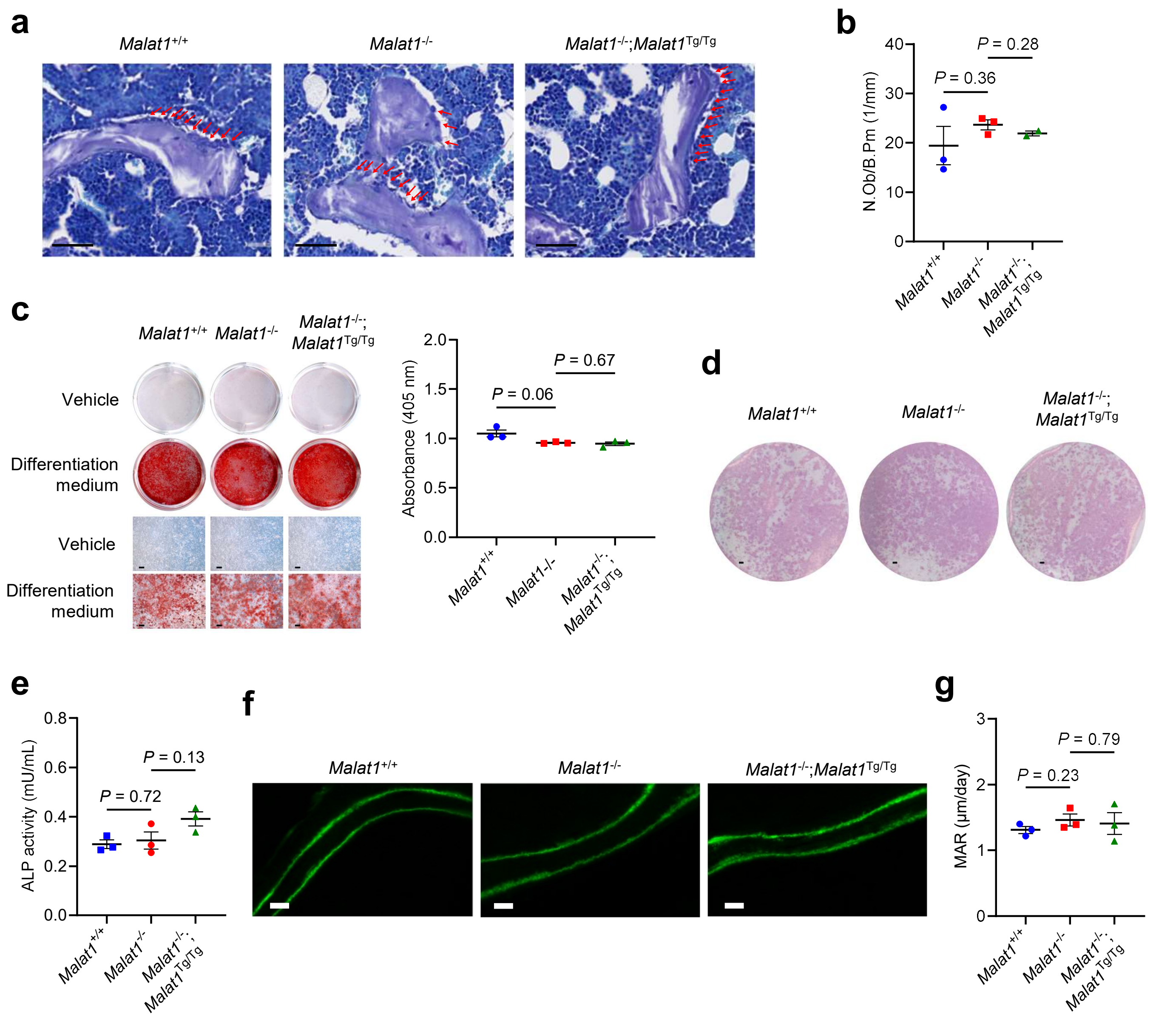

Supplementary Figure 3. Malat1 is not involved in osteoblastic bone formation.

a. Toluidine blue staining of sections of the femurs from 2-month-old male *Malat1*$^{+/+}$, *Malat1*$^{-/-}$, and *Malat1*$^{-/-}$;*Malat1*$^{Tg/Tg}$ mice. Scale bars, 50 µm. Red arrows indicate osteoblasts.

b. Quantification of the osteoblast numbers per bone perimeter (N.Ob/B.Pm) in the mice described in **a**. $n = 3$ mice per group.

c. Mesenchymal stem cells (MSCs) were isolated from *Malat1*$^{+/+}$, *Malat1*$^{-/-}$, and *Malat1*$^{-/-}$;*Malat1*$^{Tg/Tg}$ mice, cultured in osteogenic differentiation medium for 14 days, and subjected to alizarin red S (ARS) staining (left panel). Scale bars, 100 µm. ARS stains were dissolved and quantitated as absorbance at 405 nm (right panel). $n = 3$ mice per group.

d. Mesenchymal stem cells (MSCs) were isolated from *Malat1*$^{+/+}$, *Malat1*$^{-/-}$, and *Malat1*$^{-/-}$;*Malat1*$^{Tg/Tg}$ mice, cultured in differentiation medium for 14 days, and subjected to alkaline phosphatase (ALP) staining. Scale bars, 1 mm.

e. Activity of secreted ALP was measured in the culture medium collected after the MSC differentiation described in **d.** $n = 3$ mice per group.

f. Representative images of bone formation rates of 2-month-old male *Malat1*$^{+/+}$, *Malat1*$^{-/-}$, and *Malat1*$^{-/-}$;*Malat1*$^{Tg/Tg}$ mice, as determined by sequential labeling with calcein. Scale bars, 10 µm.

g. Bone histomorphometric analysis of mineral apposition rate (MAR) in the femurs of 2-month-old male *Malat1*$^{+/+}$, *Malat1*$^{-/-}$, and *Malat1*$^{-/-}$;*Malat1*$^{Tg/Tg}$ mice. $n = 3$ mice per group.

Statistical significance in **b**, **c**, **e**, and **g** was determined by a two-tailed unpaired *t*-test. Error bars are s.e.m. Source data are provided as a Source Data file.

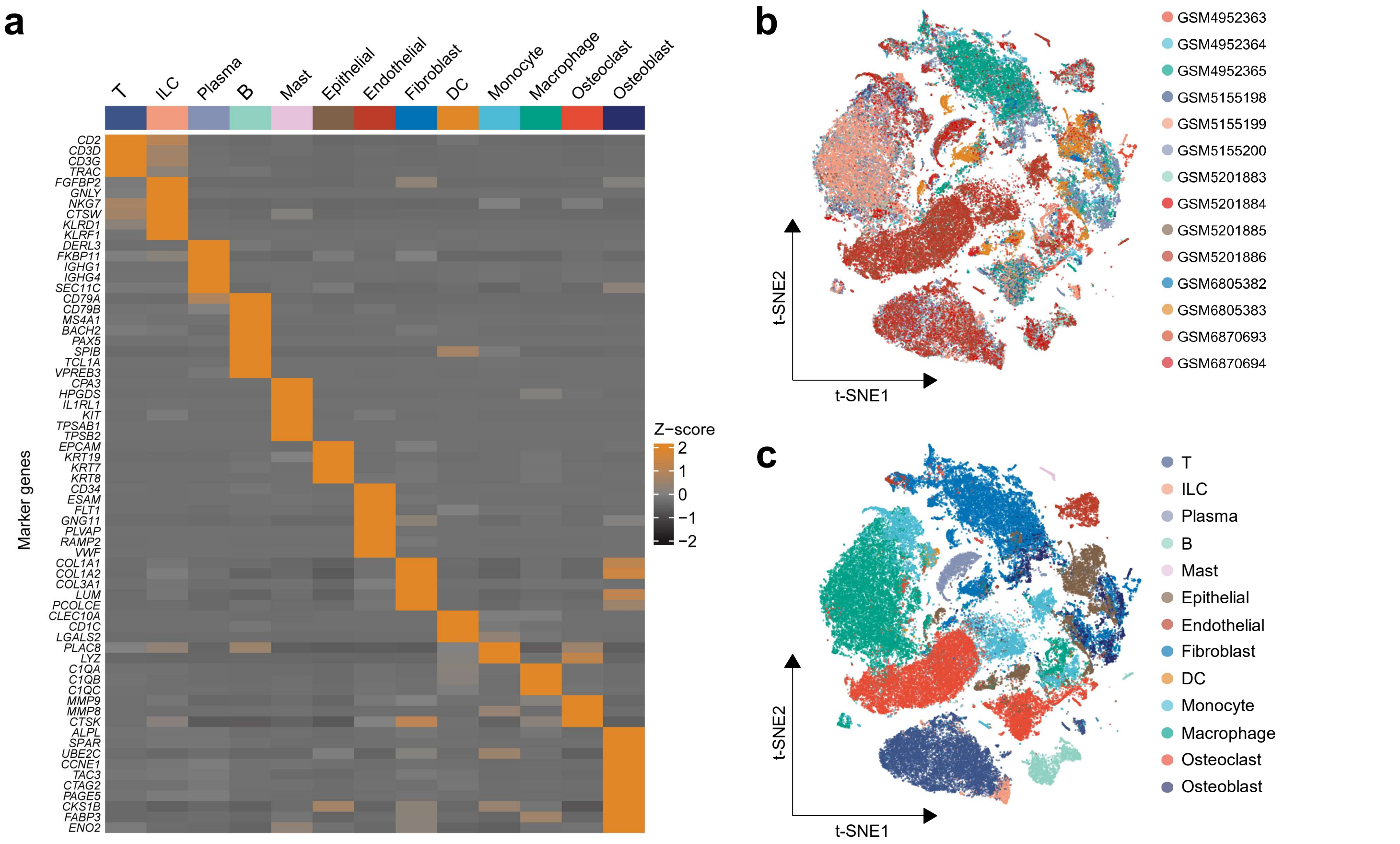

Supplementary Figure 4. The landscape of single-cell annotation.

a. Heatmap of marker genes used in the cell annotation.

b, c. The t-SNE dimensionality reduction plot (**b**) and the cell annotation t-SNE plot (**c**) showing the cellular landscape after batch effect removal by using the harmony method in the meta queue (one non-osteoporotic patient: GSM5201885; three osteoporosis patients: GSM5201883, GSM5201884, GSM5201886; six osteosarcoma patients: GSM4952363, GSM4952364, GSM4952365, GSM5155198, GSM5155199, GSM5155200; four samples from two patients with breast cancer bone metastasis: GSM6870693, GSM6870694, GSM6805382, GSM6805383).

Supplementary Figure 5

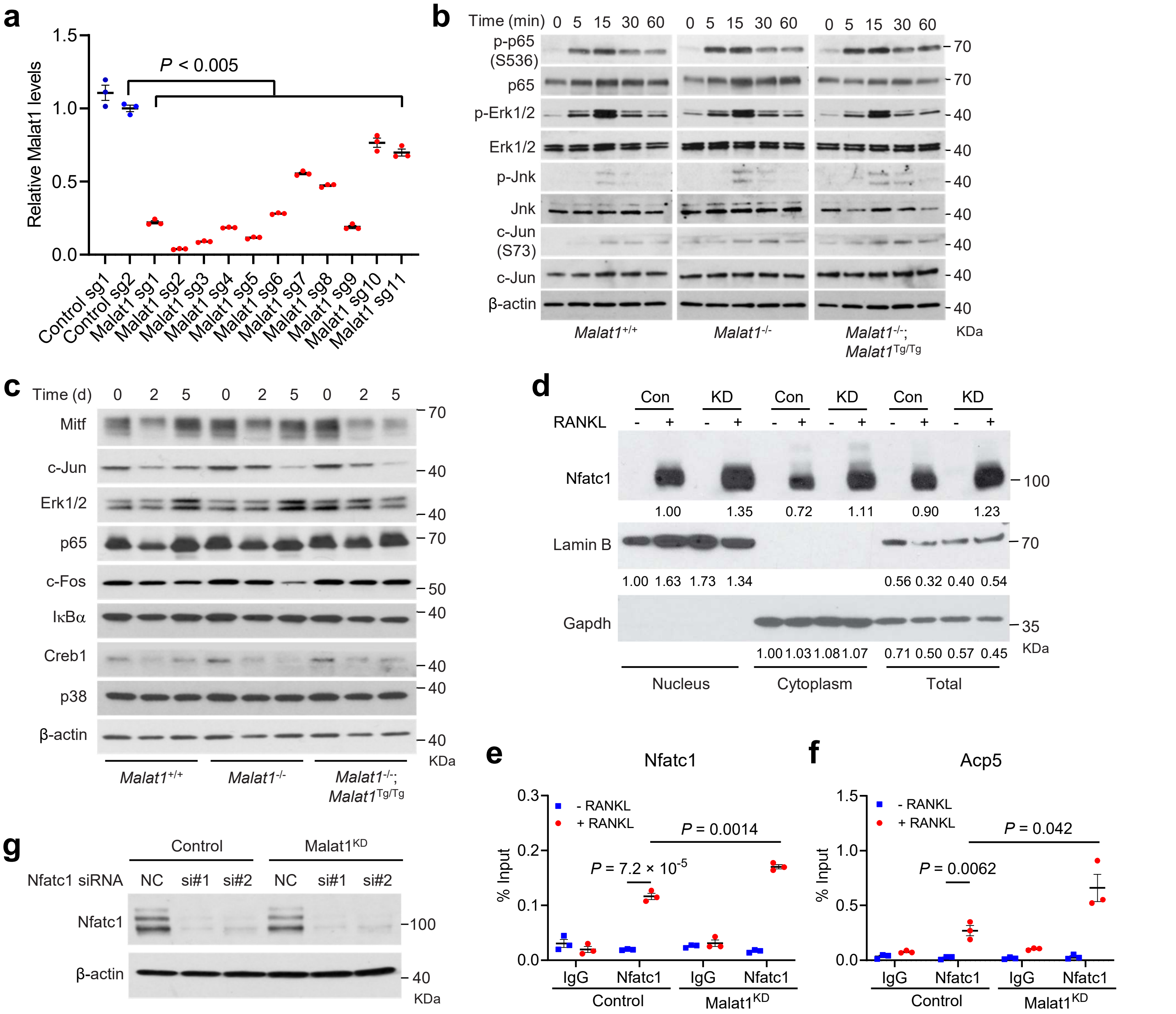

Supplementary Figure 5. Effects of CRISPRi-mediated knockdown of Malat1 on pro-osteoclastogenic signaling and Nfatc1.

a. qPCR of Malat1 in B16F1 cells with CRISPRi-mediated knockdown of Malat1. $n = 3$ biological replicates per group.

b. *Malat1*$^{+/+}$, *Malat1*$^{-/-}$, and *Malat1*$^{-/-}$;*Malat1*$^{Tg/Tg}$ BMMs were cultured with M-CSF (50 ng/mL) before stimulation with RANKL (100 ng/mL) for 0, 5, 15, 30, and 60 min. The cell lysates were subjected to immunoblotting with antibodies against phospho-p65, p65, phospho-Erk1/2, Erk1/2, phospho-Jnk, Jnk, phospho-c-Jun, c-Jun, and β-actin.

c. *Malat1*$^{+/+}$, *Malat1*$^{-/-}$, and *Malat1*$^{-/-}$;*Malat1*$^{Tg/Tg}$ BMMs were treated with M-CSF (50 ng/mL) and RANKL (100 ng/mL) for 2 days and 5 days. The cell lysates were subjected to immunoblotting with antibodies against Mitf, c-Jun, Erk1/2, p65, c-Fos, IκBα, Creb1, p38, and β-actin.

d. Control and Malat1-knockdown RAW264.7 cells were cultured with RANKL (50 ng/mL) and fractionated. The cytoplasmic and nuclear fractions, along with total cell lysates, were subjected to immunoblotting with antibodies against Nfatc1, Gapdh (a cytoplasmic marker), and Lamin B (a nuclear marker).

e, f. ChIP-qPCR analysis showing the occupancy of *Nfatc1* (**e**) and *Acp5* (**f**) gene promoters by Nfatc1 immunoprecipitated from control or Malat1-knockdown RAW264.7 cells treated with RANKL (50 ng/mL) for 3 days. $n = 3$ biological replicates per group.

g. Immunoblotting of Nfatc1 and β-actin in control and Malat1-knockdown RAW264.7 cells transfected with Nfatc1 siRNA or scrambled negative control (NC).

Statistical significance in **a**, **e**, and **f** was determined by a two-tailed unpaired *t*-test. Error bars are s.e.m. The experiments in **b-d** and **g** were repeated independently three times, yielding similar results. Source data are provided as a Source Data file.

Supplementary Figure 6

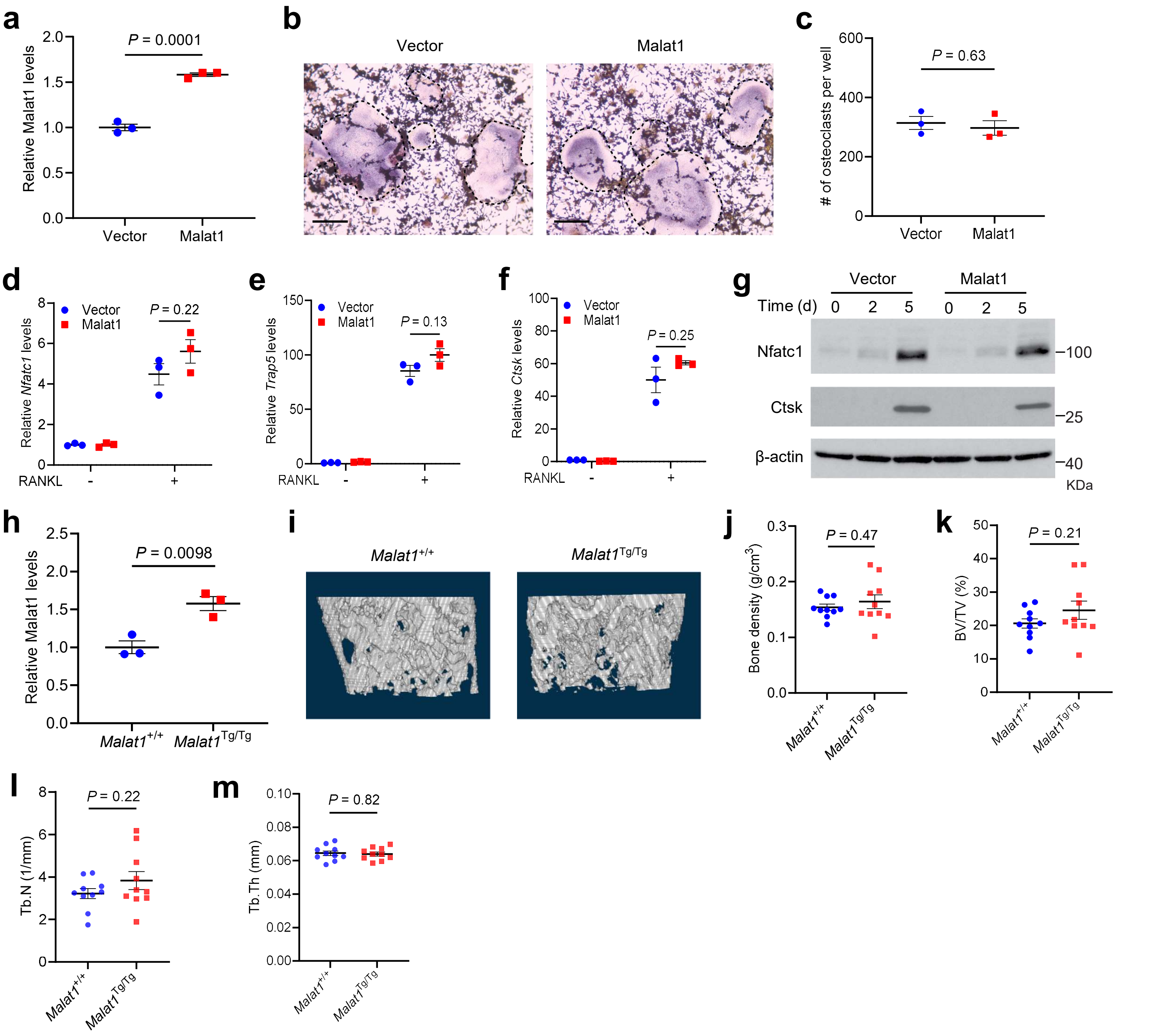

Supplementary Figure 6. Evaluation of ectopic Malat1 expression in wild-type cells and mice.

a. qPCR of Malat1 in RAW264.7 cells with ectopic Malat1 expression via a piggyBac transposon system. $n = 3$ biological replicates per group.

b, c. TRAP staining images (**b**) and quantification (**c**) of RAW264.7 cells with ectopic Malat1 expression. Cells were treated with RANKL (50 ng/mL) for 5 days. Multinucleated TRAP-positive cells (outlined by dashed lines) were counted. Scale bars, 100 μm. $n =$ 3 wells per group.

d-f. qPCR of *Nfatc1*(**d**), *Trap5* (**e**), and *Ctsk* (**f**) in RAW264.7 cells with ectopic Malat1 expression. Cells were treated with RANKL (50 ng/mL) for 3 days. $n = 3$ biological replicates per group.

g. Immunoblotting of Nfatc1, Ctsk, and β-actin in RAW264.7 cells with ectopic Malat1 expression. Cells were treated with RANKL (50 ng/mL) for 2 days and 5 days.

h. qPCR of Malat1 in the BMMs from *Malat1*$^{+/+}$ and *Malat1*$^{Tg/Tg}$ mice. $n = 3$ biological replicates per group.

i. Representative μCT images of 3D bone structures of the femurs from 6-month-old male *Malat1*$^{+/+}$ and *Malat1*$^{Tg/Tg}$ mice.

j-m. μCT-based measurements of the BMD (**j**), BV/TV (**k**), Tb.N (**l**), and Tb.th (**m**) of the femurs from 6-month-old male *Malat1*$^{+/+}$ and *Malat1*$^{Tg/Tg}$ mice, with left and right femurs for each mouse measured. $n = 5$ mice per group.

Statistical significance in **a**, **c-f**, **h**, and **j-m** was determined by a two-tailed unpaired *t*-test. Error bars are s.e.m. The experiments in **g** were repeated independently three times, yielding similar results. Source data are provided as a Source Data file.

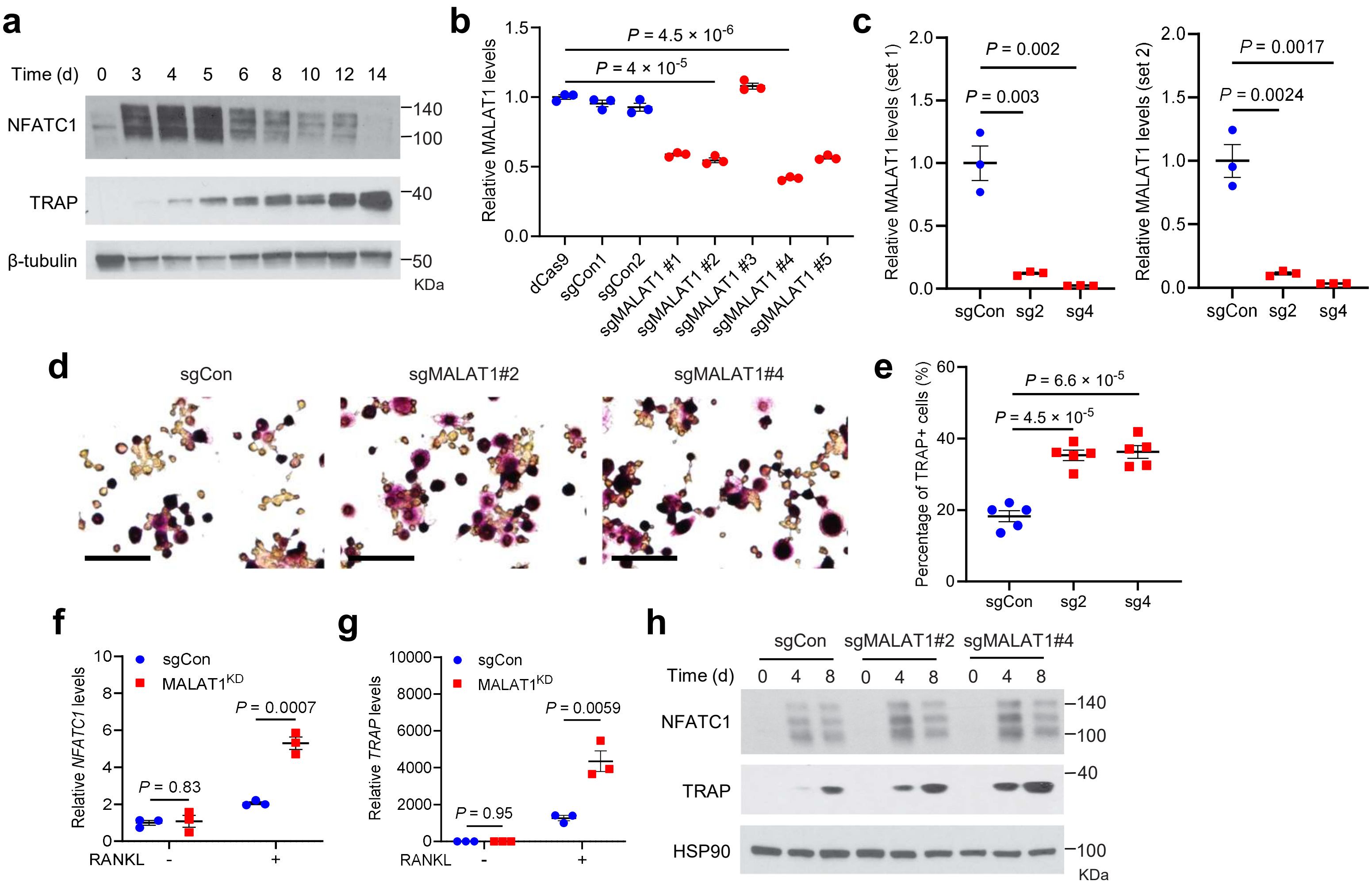

Supplementary Figure 7. MALAT1 depletion promotes osteoclast differentiation in U937 human pre-osteoclast cells.

a. Immunoblotting of NFATC1, TRAP, and β-tubulin in U937 cells treated with PMA (100 ng/mL) for 2 days, followed by treatment with M-CSF (50 ng/mL) and RANKL (100 ng/mL) for the indicated times.

b. qPCR of MALAT1 in HEK293T cells with CRISPRi-mediated knockdown of MALAT1. $n = 3$ biological replicates per group.

c. qPCR of MALAT1 in U937 cells with CRISPRi-mediated knockdown of MALAT1. Left panel: MALAT1 qPCR primer set 1; right panel: MALAT1 qPCR primer set 2. $n = 3$ biological replicates per group.

d, e. TRAP staining images (**d**) and quantification (**e**) of control and MALAT1-knockdown U937 cells treated with PMA (100 ng/mL) for 2 days, followed by treatment with M-CSF (50 ng/mL) and RANKL (100 ng/mL) for 12 days. Scale bars, 100 µm. $n = 5$ wells per group.

f, g. qPCR of *NFATC1* (**f**) and *TRAP* (**g**) in control and MALAT1-knockdown U937 cells treated with M-CSF and RANKL for 4 days. $n = 3$ biological replicates per group.

h. Immunoblotting of NFATC1, TRAP, and HSP90 in control and MALAT1-knockdown U937 cells treated with M-CSF and RANKL for 4 and 8 days.

Statistical significance in **b**, **c**, and **e-g** was determined by a two-tailed unpaired *t*-test. Error bars are s.e.m. The experiments in **a** and **h** were repeated independently three times, yielding similar results. Source data are provided as a Source Data file.

Supplementary Figure 8

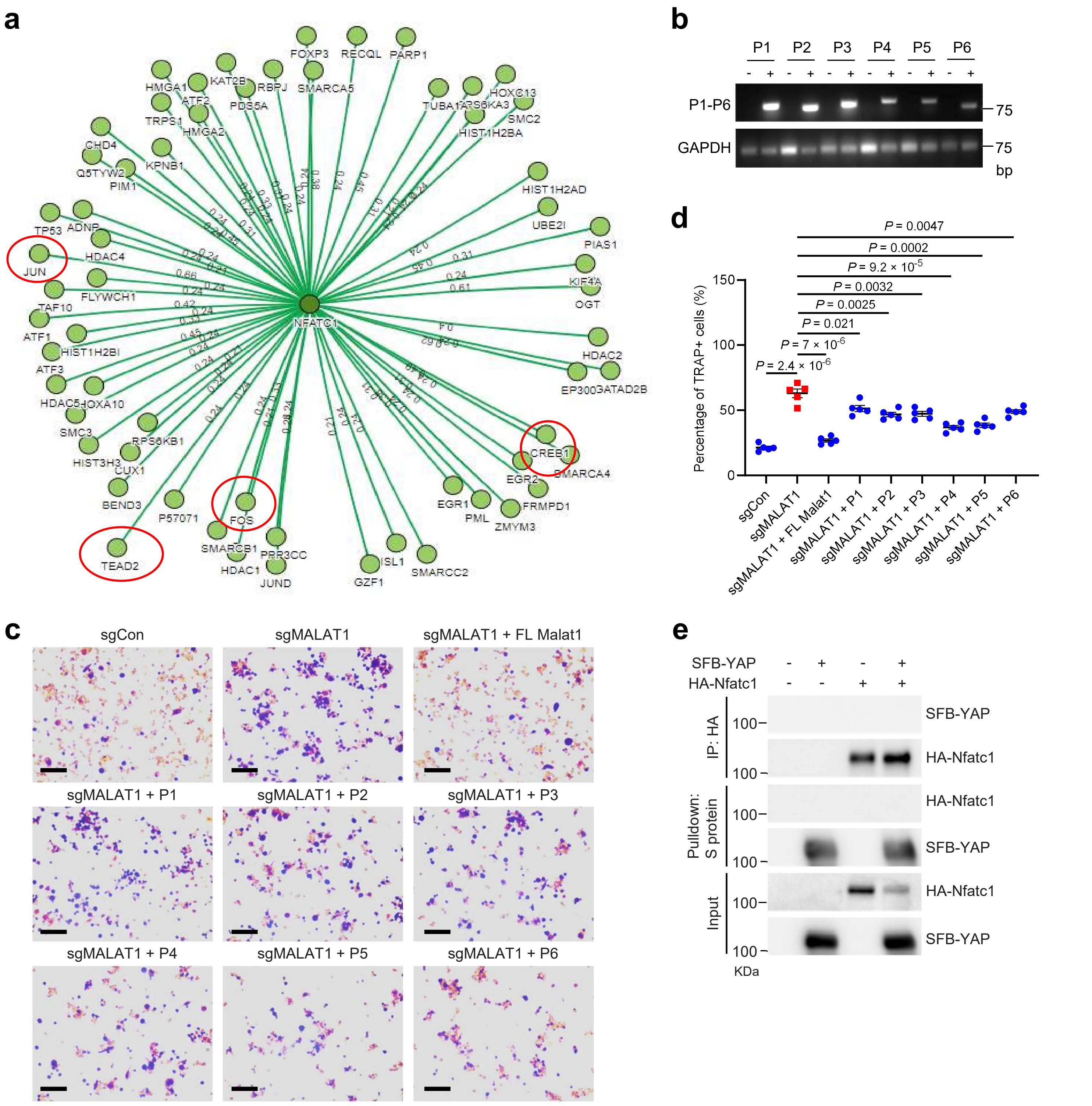

Supplementary Figure 8. Analysis of the interaction of NFATC1 with other proteins and functional analysis of full-length Malat1 and Malat1 fragments

a. Schematic representation of NFATC1-binding proteins, based on the Mentha database (http://mentha.uniroma2.it/index.php). The threshold value was an evidence score of > 0.2.

b. RT-PCR of Malat1 fragments (P1-P6) in MALAT1-depleted U937 cells with re-expression of Malat1 fragments.

c, d. TRAP staining images (**c**) and quantification (**d**) of control, MALAT1-knockdown, and Malat1-rescued (with either full-length Malat1 or Malat1 fragments) U937 cells treated with PMA (100 ng/mL) for 2 days, followed by treatment with M-CSF (50 ng/mL) and RANKL (100 ng/mL) for 12 days. Scale bars in **c**, 100 µm. Statistical significance in **d** was determined by a two-tailed unpaired *t*-test. Error bars are s.e.m. $n = 5$ wells per group.

e. HEK293T cells were co-transfected with HA-Nfatc1 and SFB-YAP and subjected to pulldown with an HA-specific antibody or S-protein beads, followed by immunoblotting with antibodies against FLAG and HA.

The experiments in **b** and **e** were repeated independently three times, yielding similar results. Source data are provided as a Source Data file.

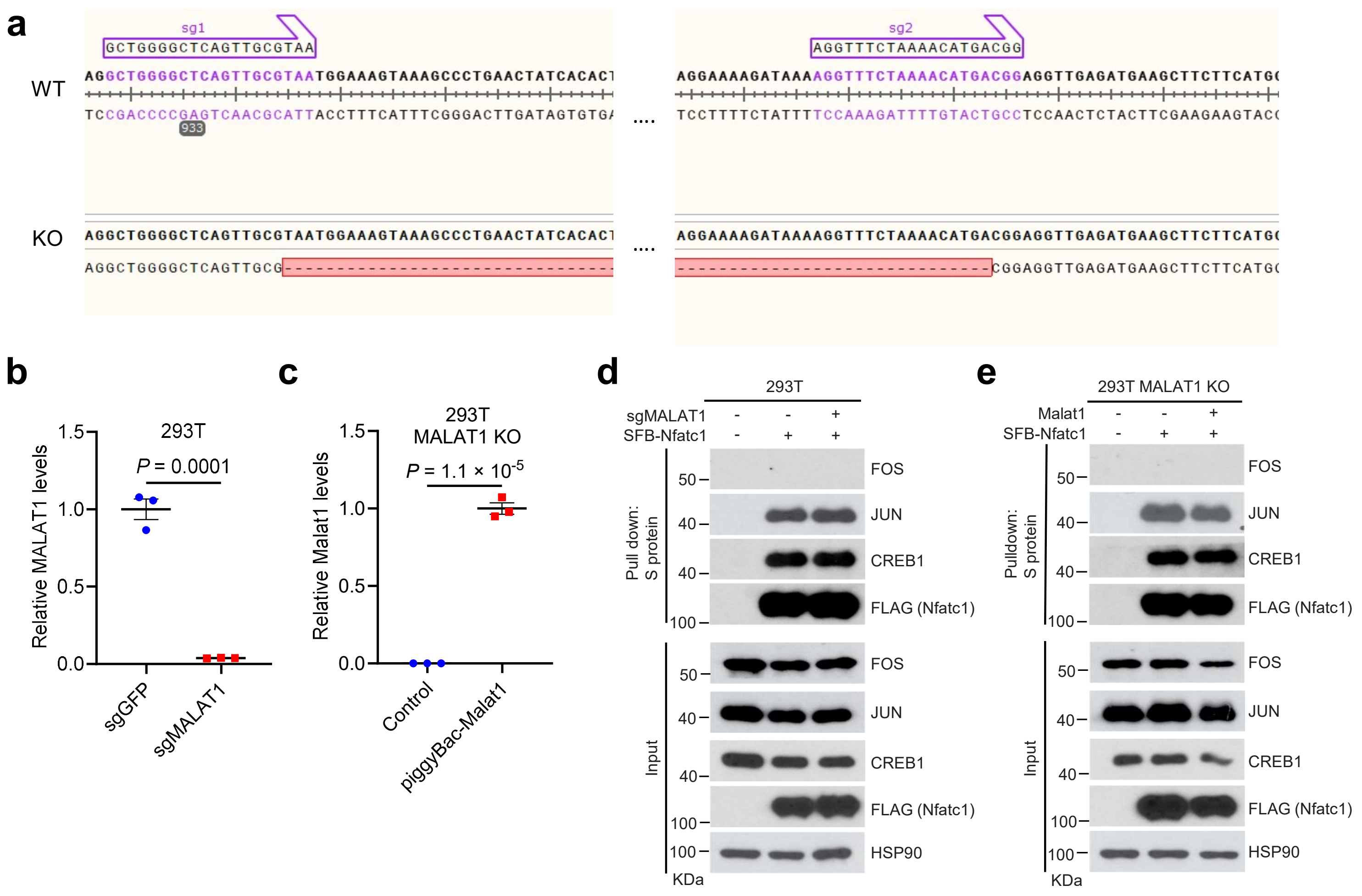

Supplementary Figure 9. MALAT1 has no effect on the interaction of NFATC1 with other transcription regulators involved in osteoclastogenesis.

a. Schematic representation of the generation of MALAT1-null HEK293T cells by using a double excision CRISPR-knockout method. The MALAT1-knockout cells were verified by DNA sequencing as lacking the fragment between the two sgRNA sites.

b. qPCR of MALAT1 in control and MALAT1-knockout HEK293T cells. $n = 3$ biological replicates per group.

c. qPCR of Malat1 in MALAT1-knockout HEK293T cells with or without piggyBac-mediated expression of Malat1. $n = 3$ biological replicates per group.

d. Control and MALAT1-knockout HEK293T cells transfected with SFB-Nfatc1 were subjected to pulldown with S-protein beads, followed by immunoblotting with antibodies against FOS, JUN, CREB1, FLAG, and HSP90.

e. MALAT1-knockout and Malat1-restored HEK293T cells co-transfected with SFB-Nfatc1 were subjected to pulldown with S-protein beads, followed by immunoblotting with antibodies against FOS, JUN, CREB1, FLAG, and HSP90.

Statistical significance in **b** and **c** was determined by a two-tailed unpaired *t*-test. Error bars are s.e.m. The experiments in **d** and **e** were repeated independently three times, yielding similar results. Source data are provided as a Source Data file.

Supplementary Figure 10

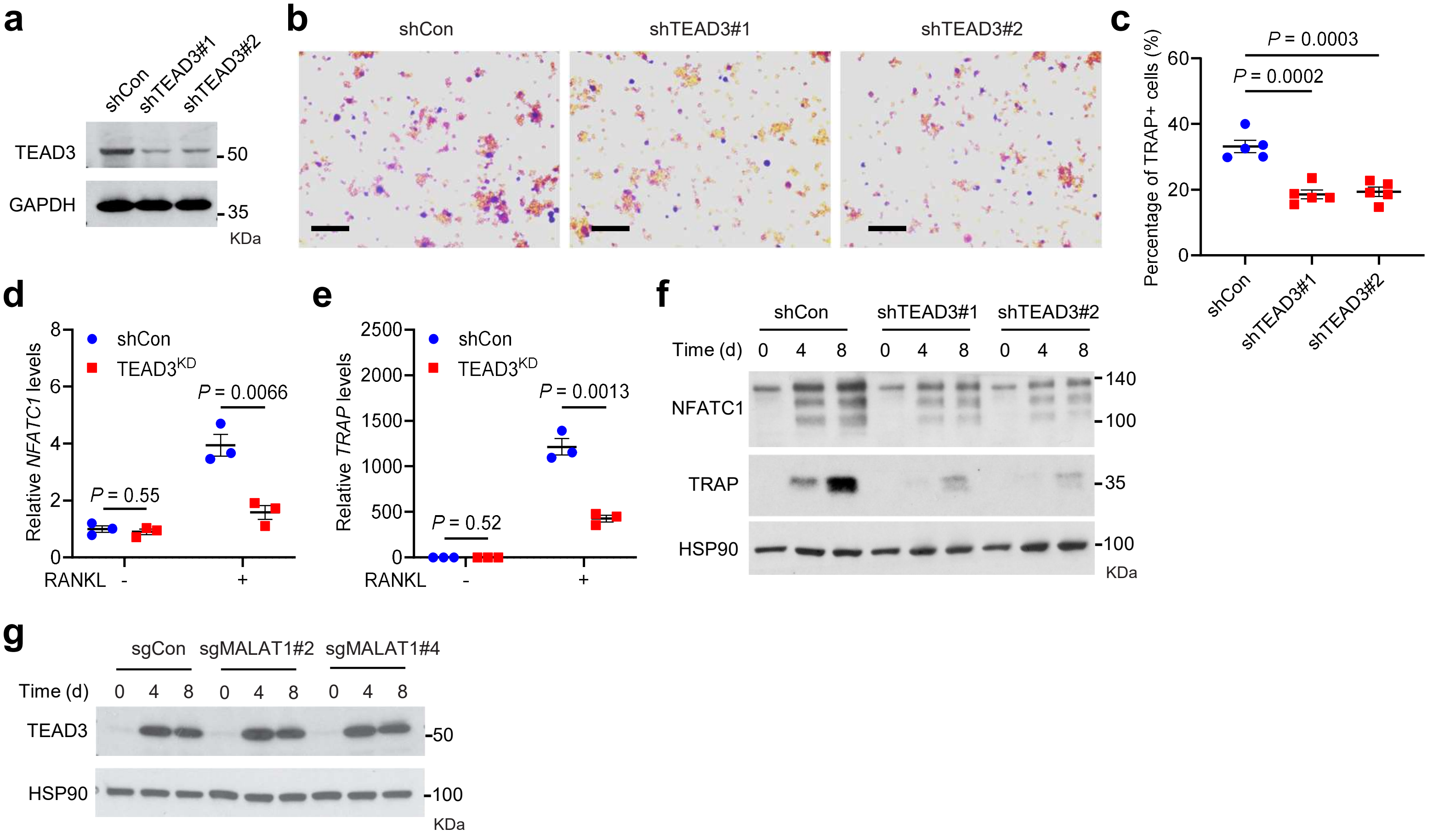

Supplementary Figure 10. Effect of TEAD3 knockdown on RANKL-induced human osteoclastogenesis.

a. Immunoblotting of TEAD3 and HSP90 in U937 cells transduced with two independent TEAD3 shRNAs or scrambled control shRNA.

b, c. TRAP staining images (**b**) and quantification (**c**) of control and TEAD3-knockdown U937 cells treated with M-CSF and RANKL for 14 days. *n* = 5 wells per group.

d, e. qPCR of NFATC1(**d**) and TRAP (**e**) in control and TEAD3-knockdown U937 cells treated with M-CSF and RANKL for 4 days. *n* = 3 biological replicates per group.

f. Immunoblotting of NFATC1, TRAP, and HSP90 in control and TEAD3-knockdown U937 cells treated with M-CSF and RANKL for 4 days and 8 days.

g. Immunoblotting of TEAD3 and HSP90 in control and MALAT1-knockdown U937 cells treated with M-CSF and RANKL for 4 days and 8 days.

Statistical significance in **c-e** was determined by a two-tailed unpaired *t*-test. Error bars are s.e.m. The experiments in **a**, **f**, and **g** were repeated independently three times, yielding similar results. Source data are provided as a Source Data file.

Supplementary Figure 11

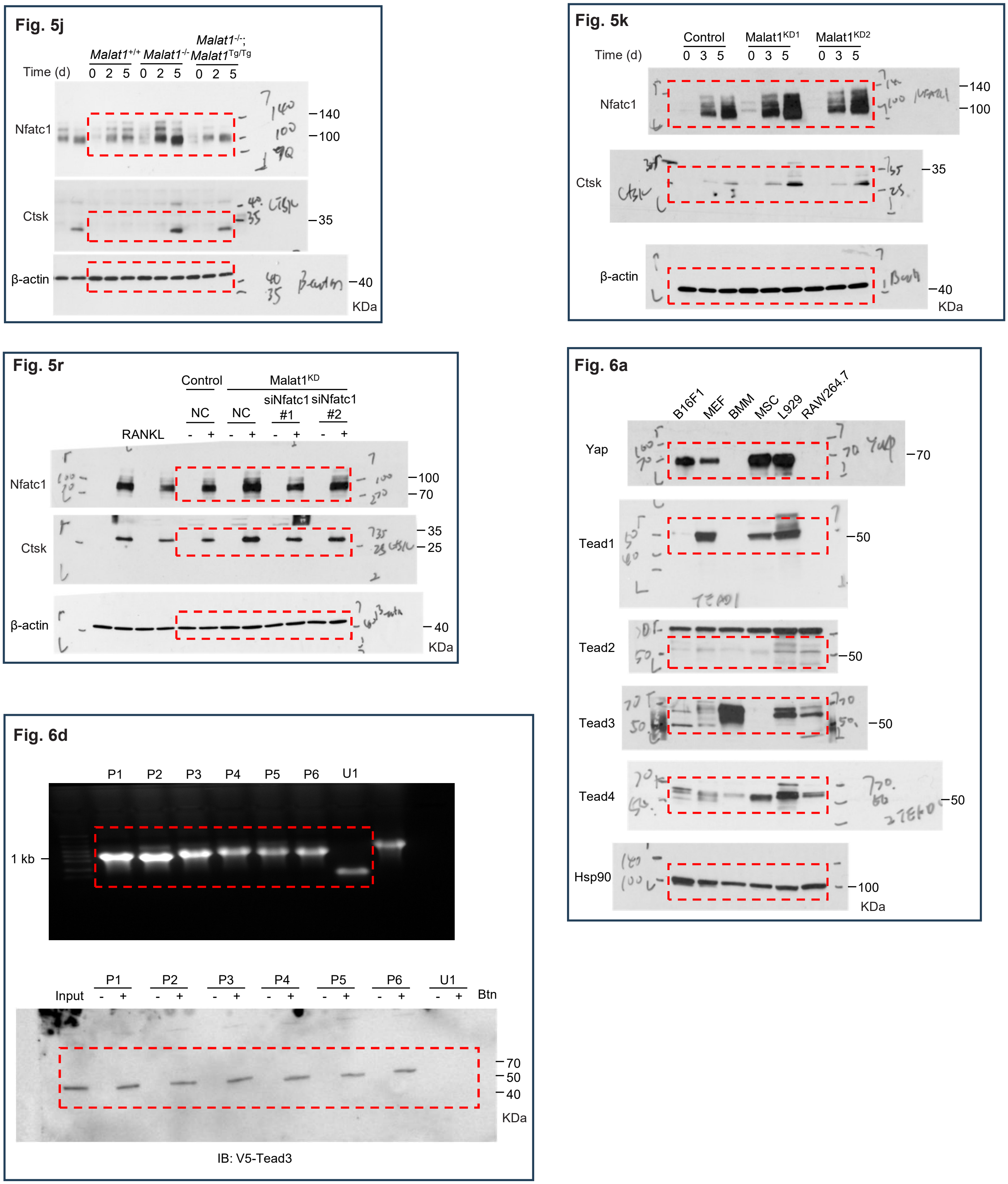

Supplementary Figure 11. All uncropped blots and gels in this manuscript.

Supplementary Figure 11

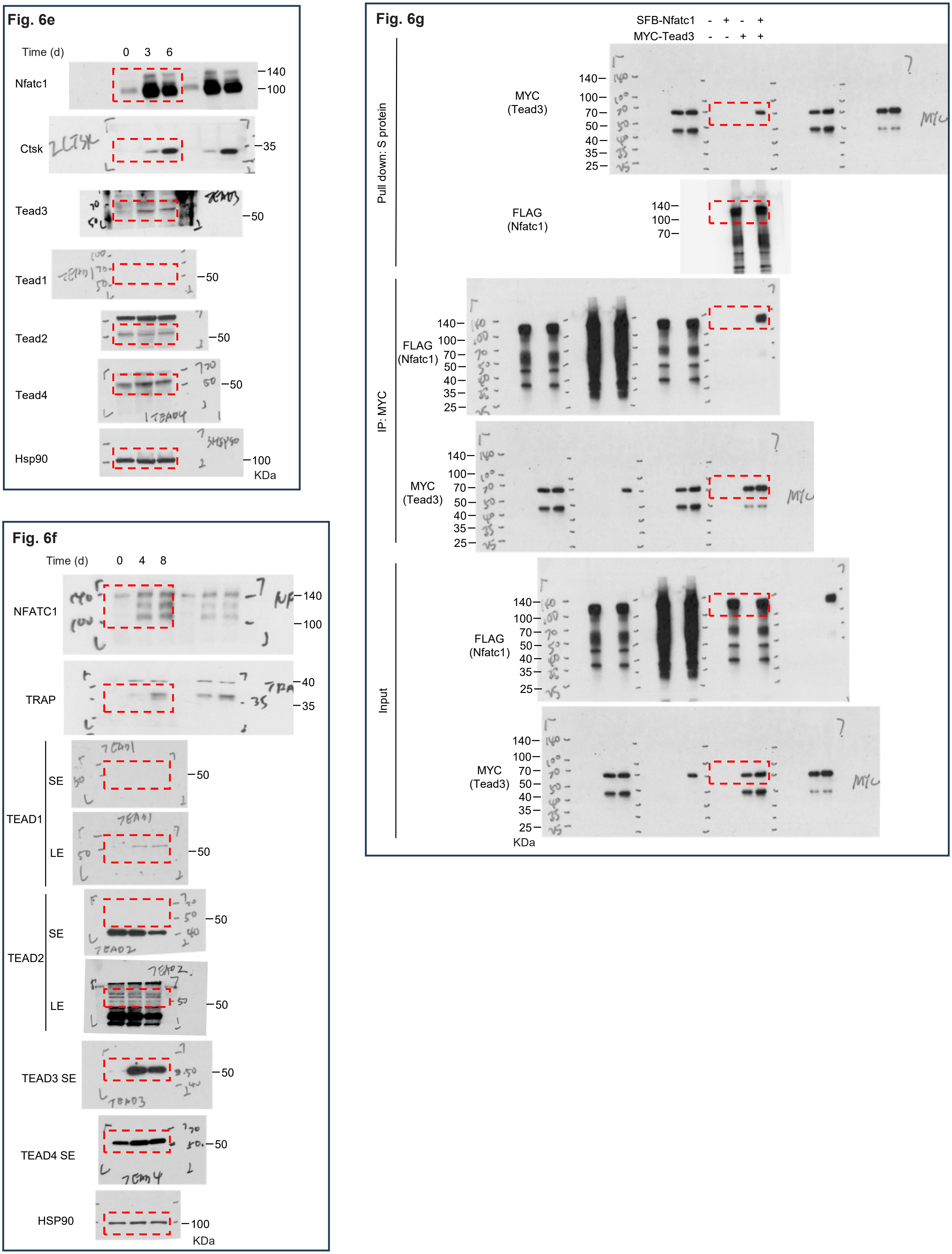

Supplementary Figure 11. All uncropped blots and gels in this manuscript.

Supplementary Figure 11

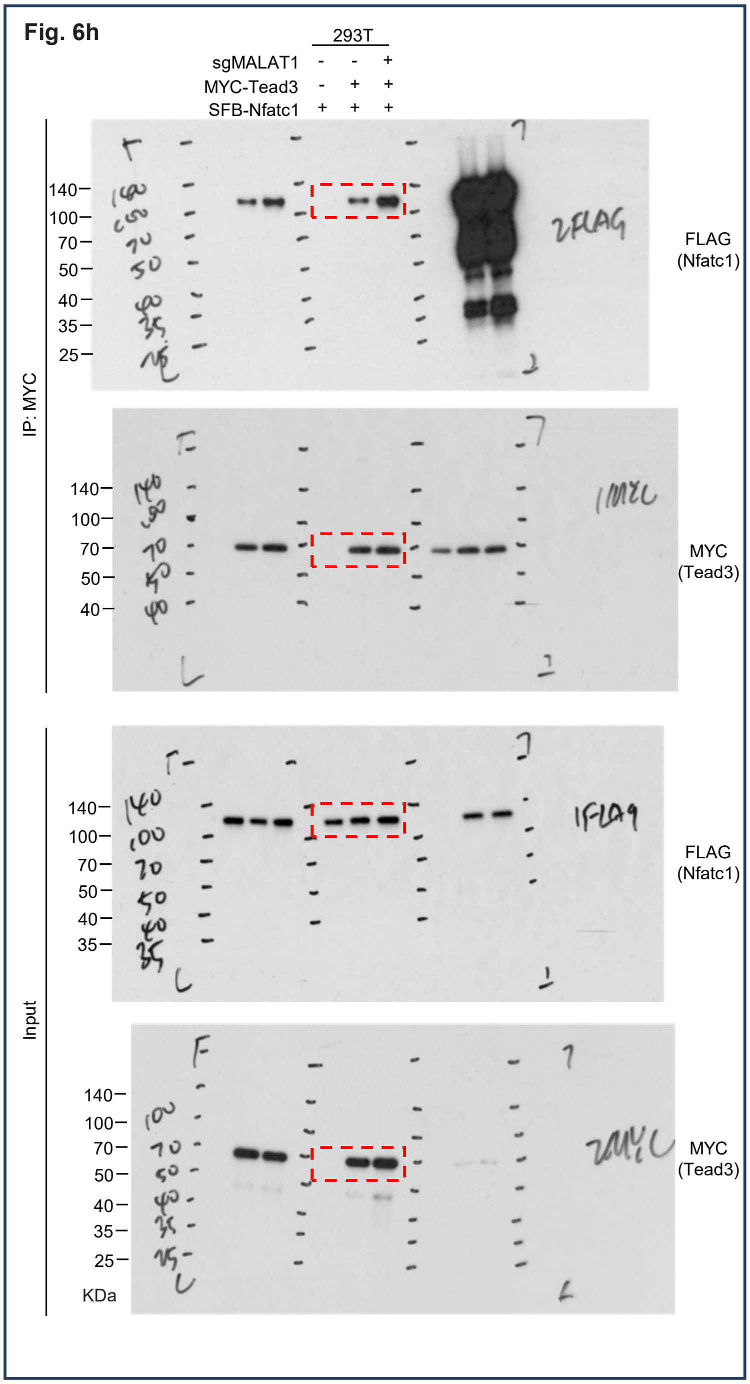

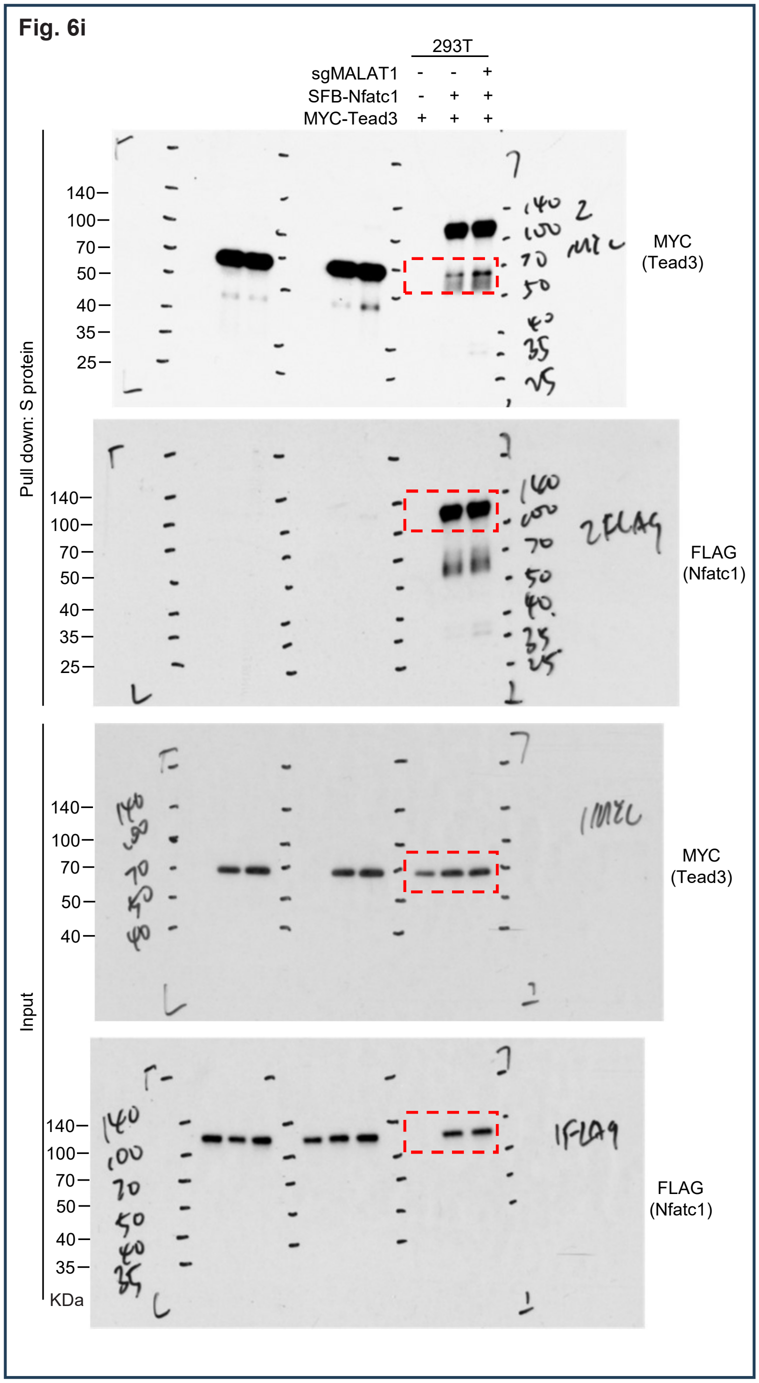

Supplementary Figure 11. All uncropped blots and gels in this manuscript.

Supplementary Figure 11

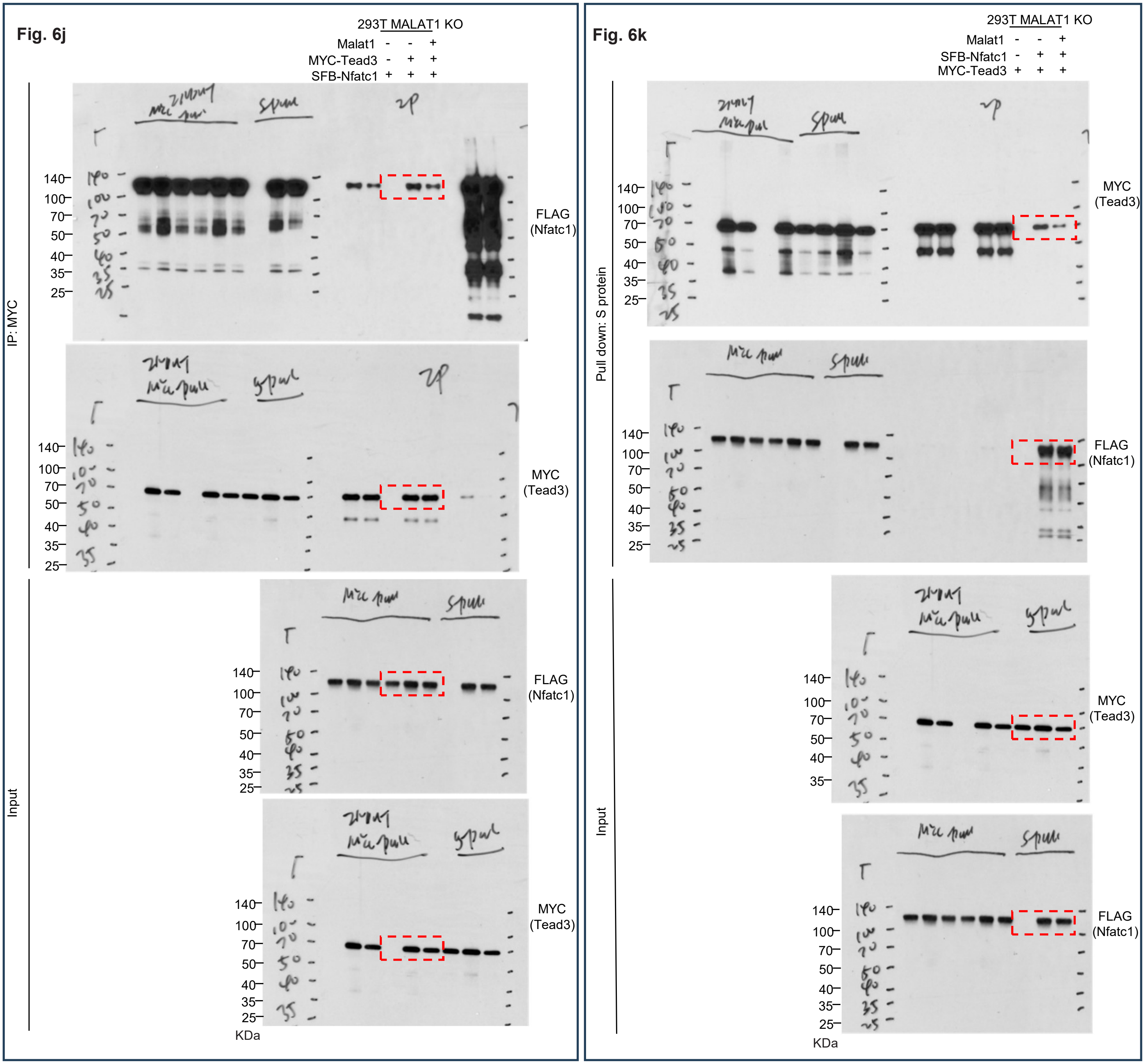

Supplementary Figure 11. All uncropped blots and gels in this manuscript.

Supplementary Figure 11

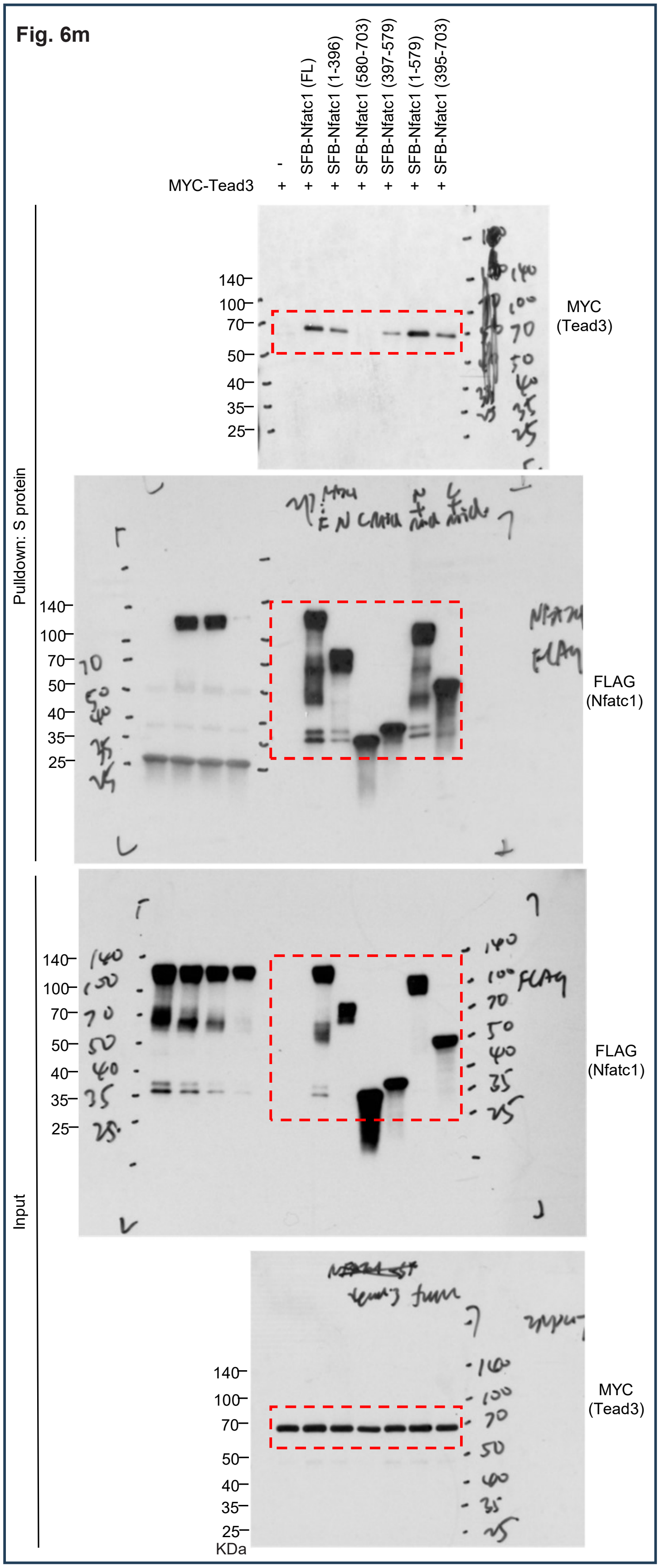

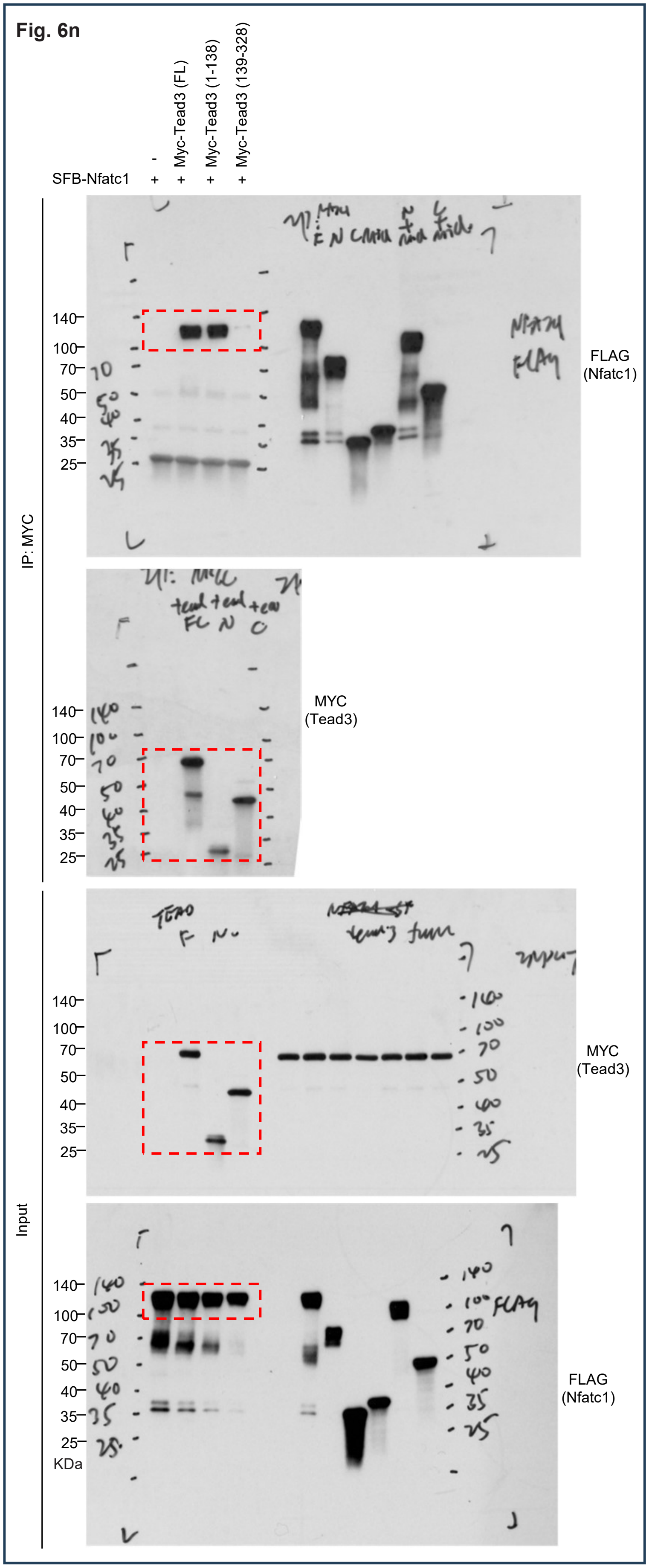

Supplementary Figure 11. All uncropped blots and gels in this manuscript.

Supplementary Figure 11

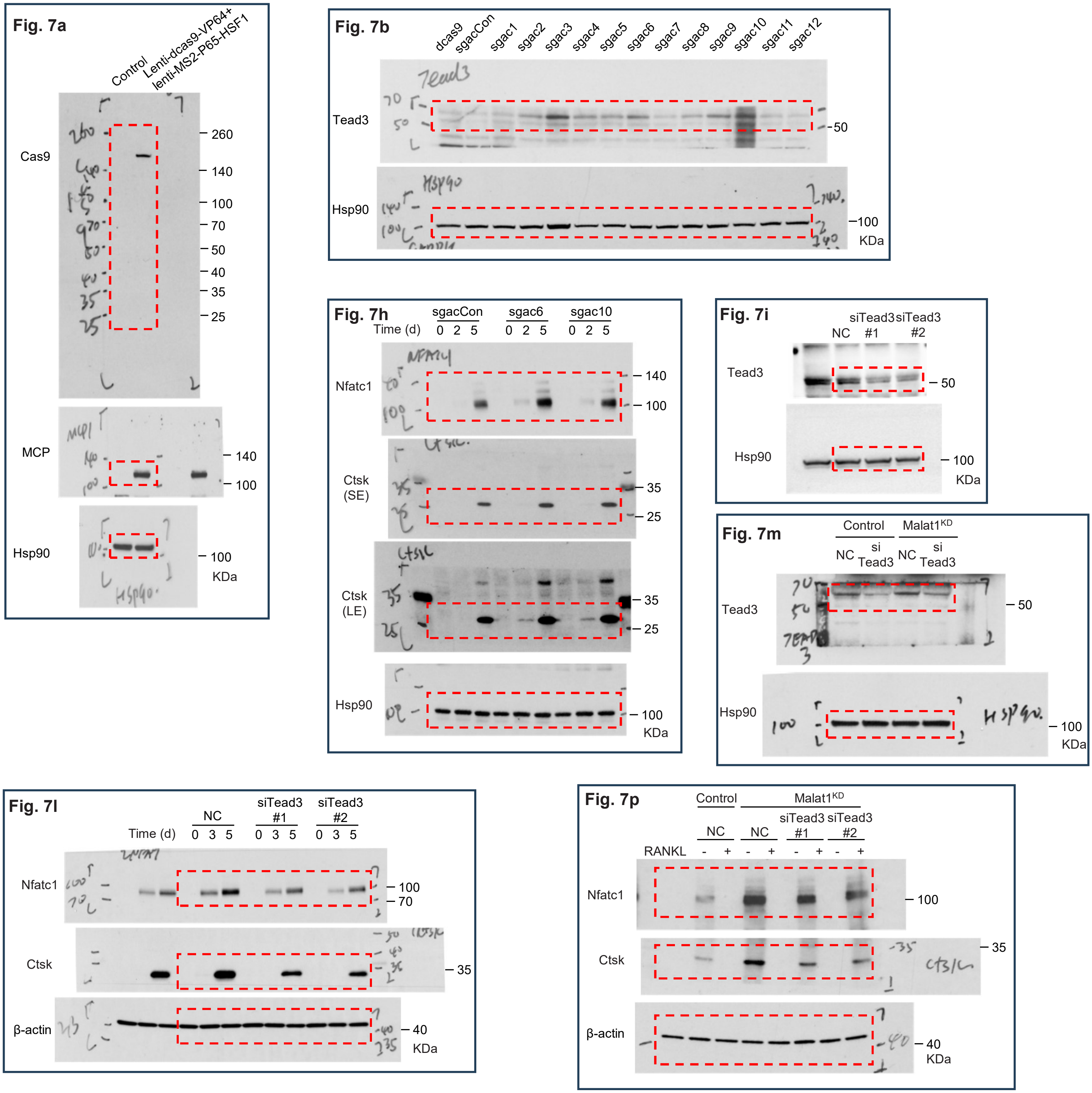

Supplementary Figure 11. All uncropped blots and gels in this manuscript.

Supplementary Figure 11

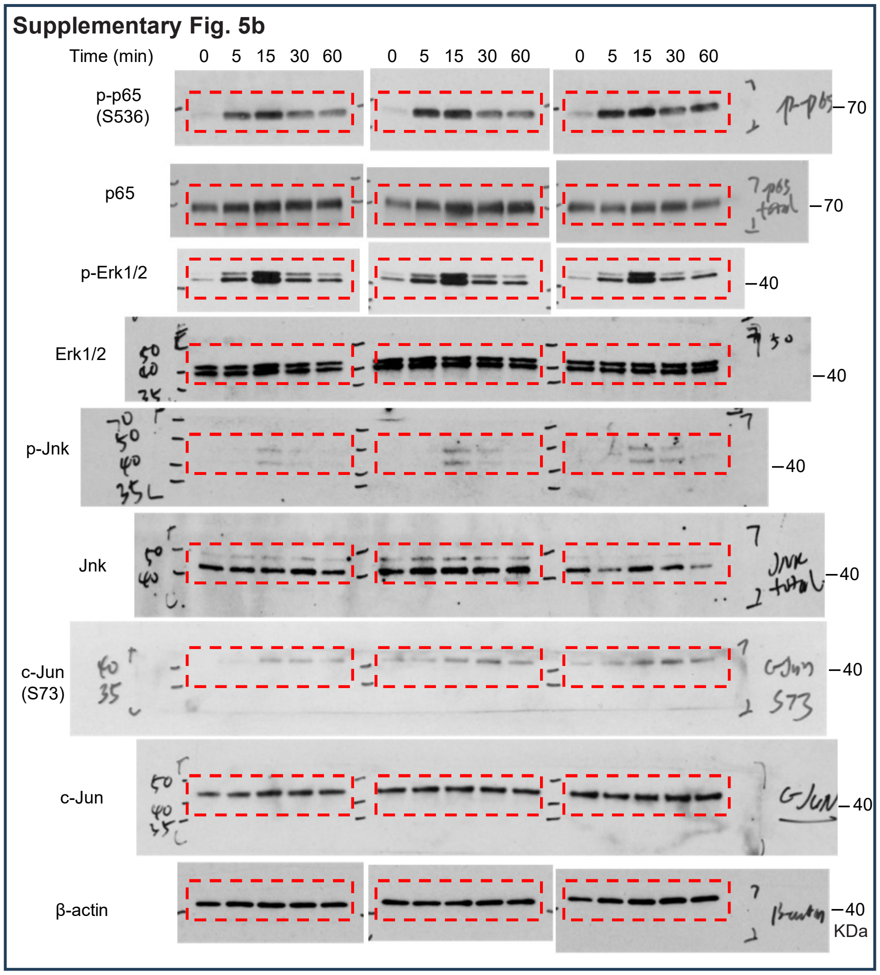

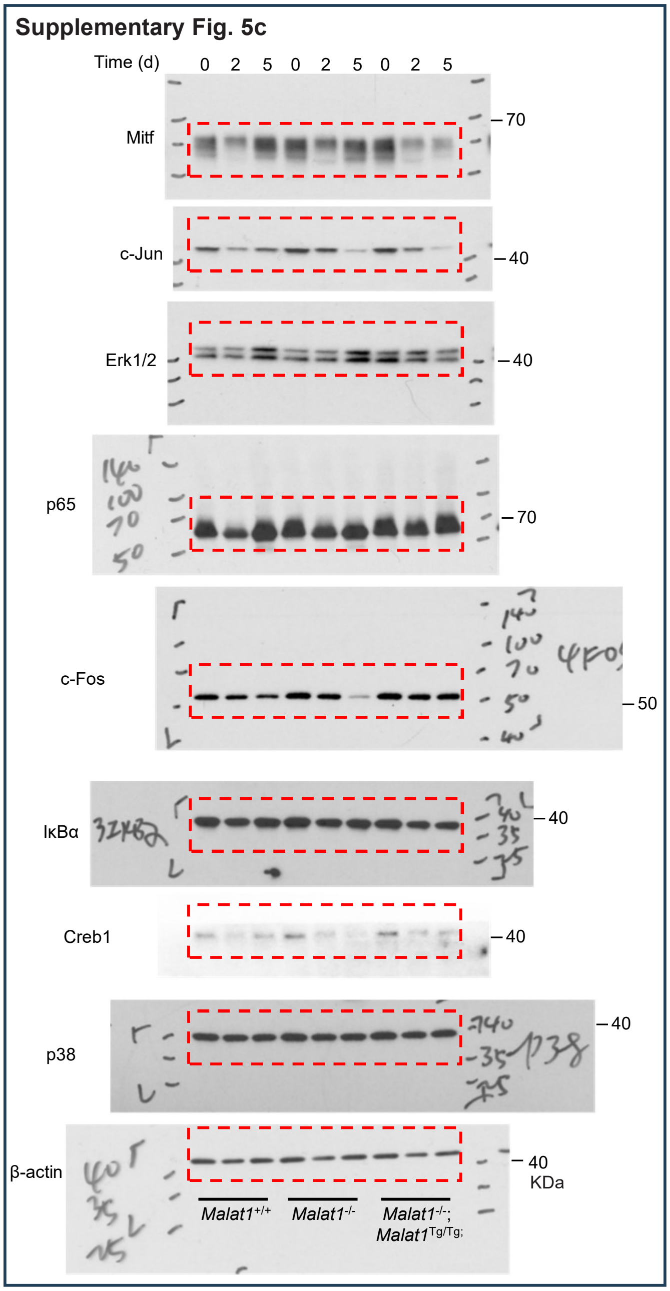

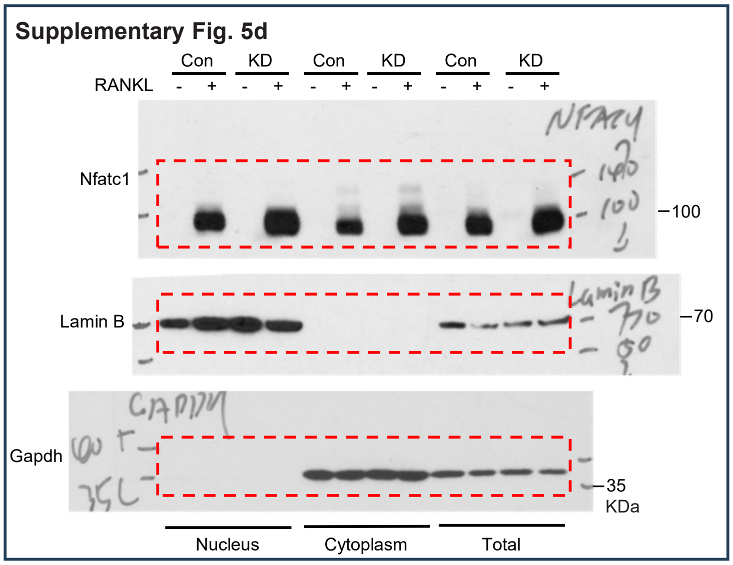

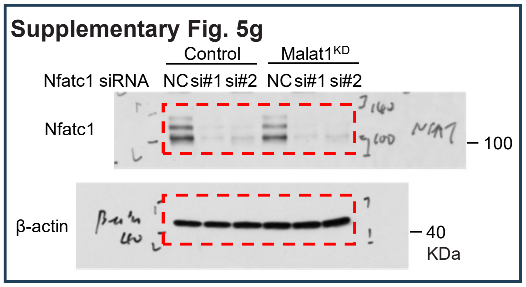

Supplementary Figure 11. All uncropped blots and gels in this manuscript.

Supplementary Figure 11

Supplementary Fig. 6g

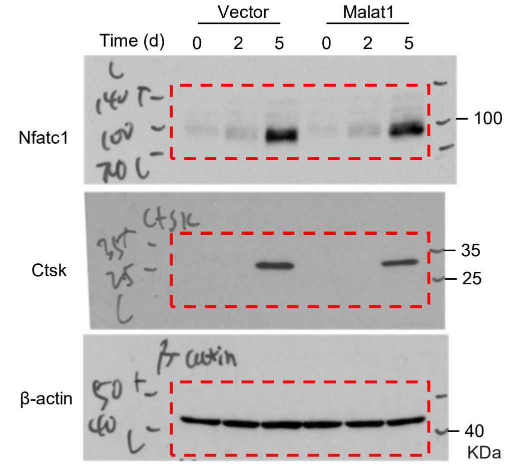

Supplementary Fig. 7a

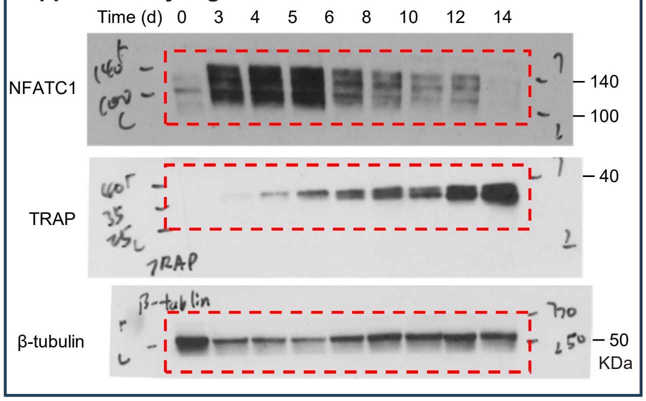

Supplementary Fig. 7h

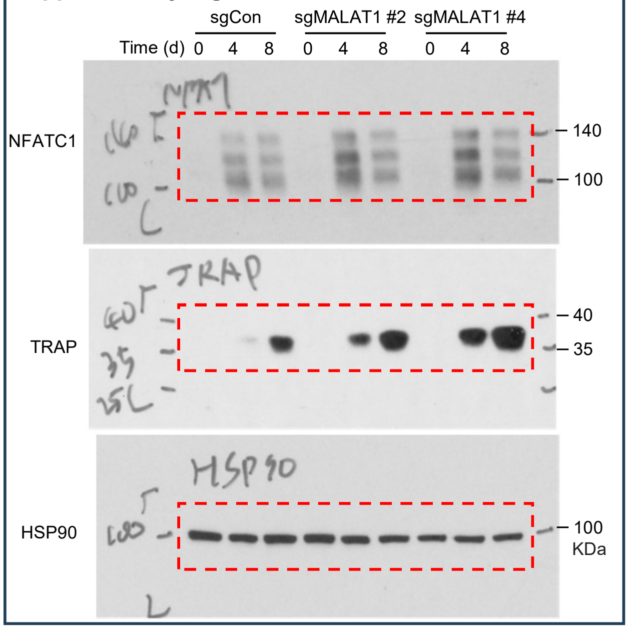

Supplementary Fig. 8b

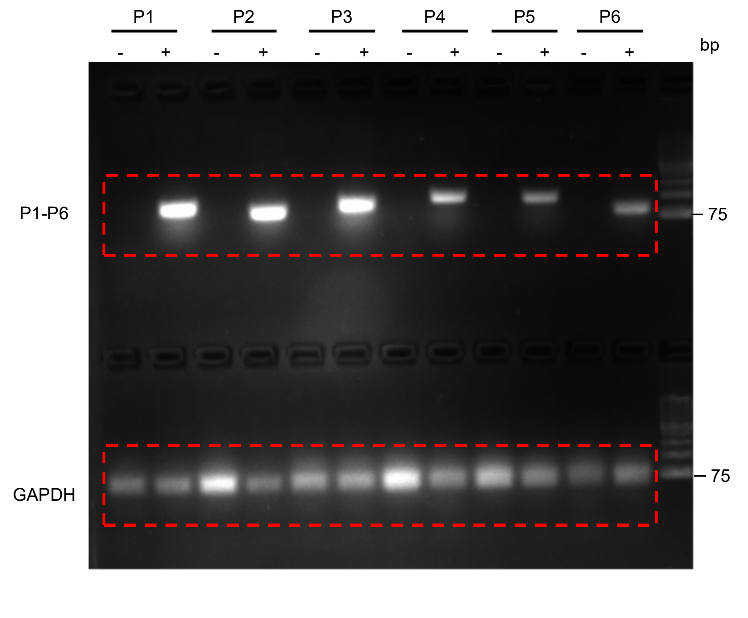

Supplementary Fig. 8e

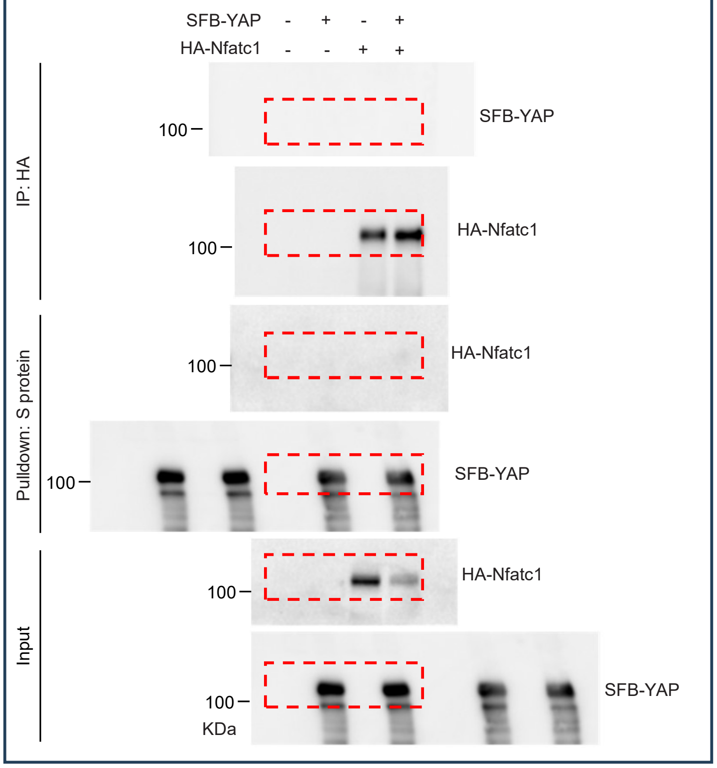

Supplementary Figure 11.
All uncropped blots and gels in this manuscript.

Supplementary Figure 11

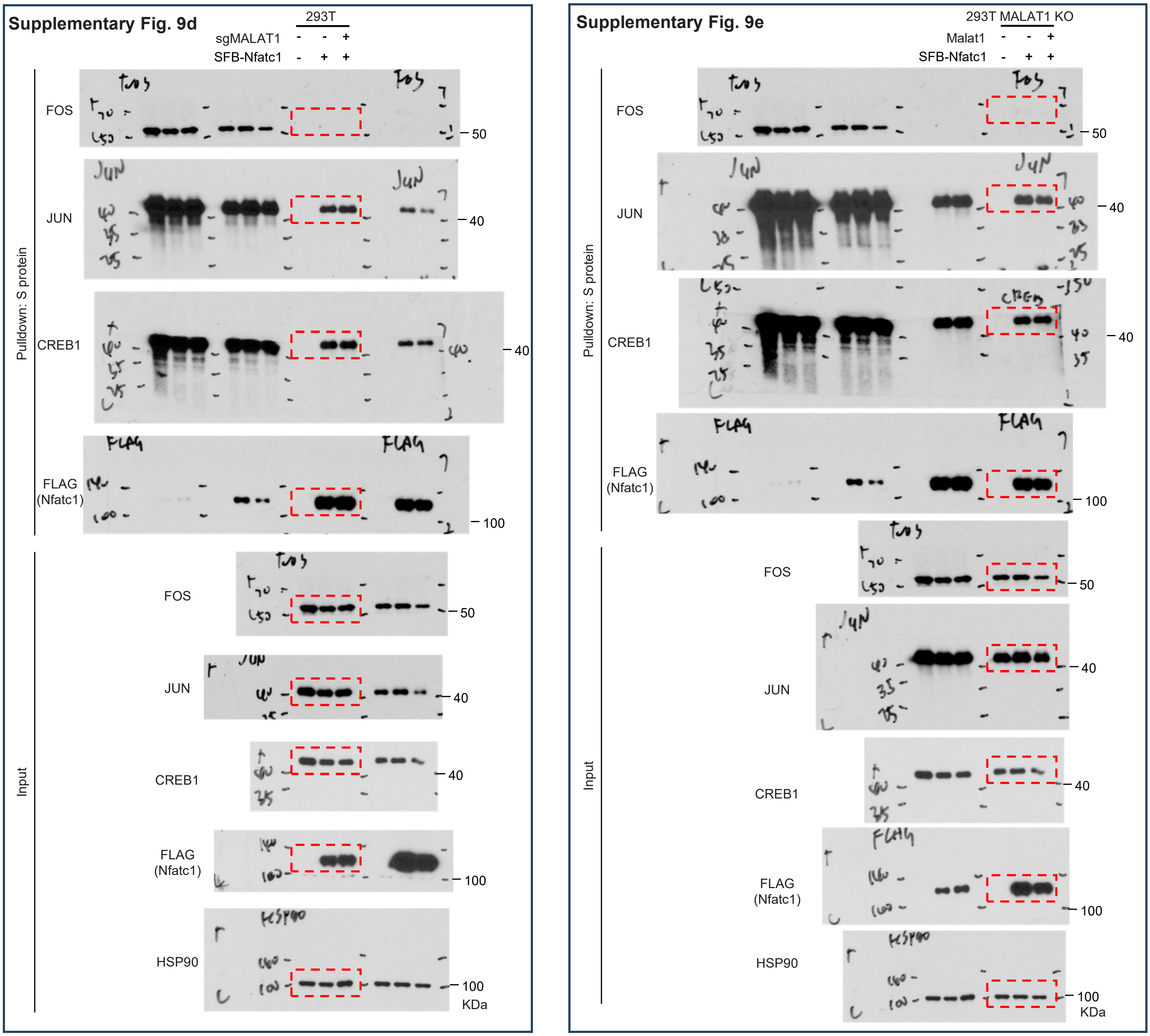

Supplementary Figure 11. All uncropped blots and gels in this manuscript.

Supplementary Figure 11

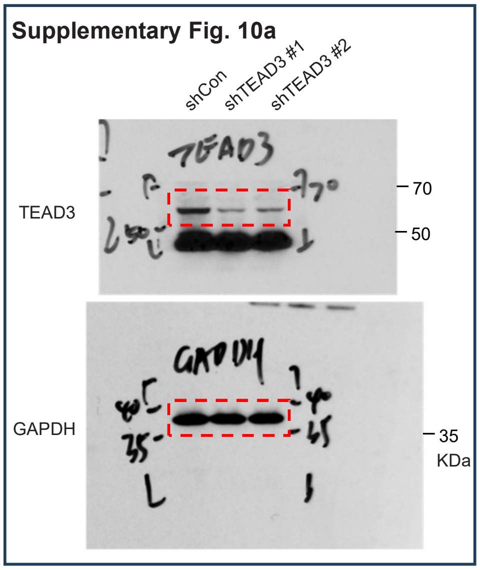

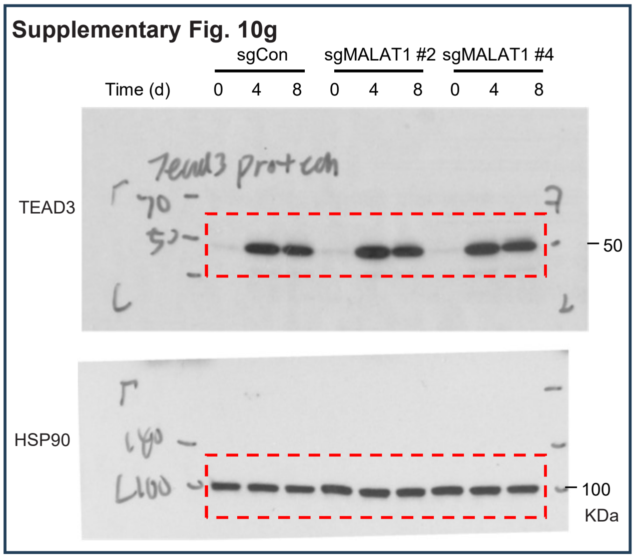

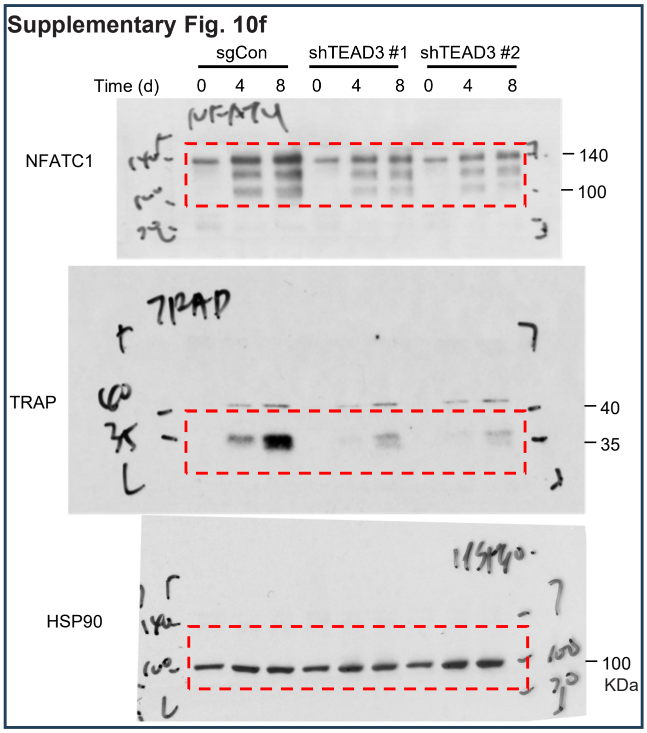

Supplementary Figure 11. All uncropped blots and gels in this manuscript.
