# Supplementary material for: Long noncoding RNA Malat1 protects against osteoporosis and bone metastasis
Source: Nat Commun. 2024 Mar 16;15:2384. doi: 10.1038/s41467-024-46602-3 (PMC10944492; doi:10.1038/s41467-024-46602-3)
Supplement: Supplementary file 3 — Description of Additional Supplementary Files [file 41467_2024_46602_MOESM3_ESM.pdf]

**Description of Additional Supplementary Files**

**File Name: Supplementary Data 1**
**Description:** Gene set enrichment analysis of human CD14+ macrophages and MGCs. NES: normalized enrichment score. p.adjust < 0.05.

**File Name: Supplementary Data 2**
**Description:** Primers used for cloning.

**File Name: Supplementary Data 3**
**Description:** sgRNA sequences for CRISPRi and CRISPRa.

**File Name: Supplementary Data 4**
**Description:** Primer sequences for qPCR.

**File Name: Supplementary Data 5**
**Description:** Primer sequences for ChIP-qPCR.
