# Supplementary material for: Long noncoding RNA Malat1 protects against osteoporosis and bone metastasis
Source: Nat Commun. 2024 Mar 16;15:2384. doi: 10.1038/s41467-024-46602-3 (PMC10944492; doi:10.1038/s41467-024-46602-3)
Supplement: Supplementary file 5 — Reporting Summary [file 41467_2024_46602_MOESM5_ESM.pdf]

nature portfolio

Corresponding author(s): Li Ma

Last updated by author(s): Feb 14, 2024

Reporting Summary

Nature Portfolio wishes to improve the reproducibility of the work that we publish. This form provides structure for consistency and transparency in reporting. For further information on Nature Portfolio policies, see our Editorial Policies and the Editorial Policy Checklist.

Statistics

For all statistical analyses, confirm that the following items are present in the figure legend, table legend, main text, or Methods section.

n/a	Confirmed	
☐	☒	The exact sample size (n) for each experimental group/condition, given as a discrete number and unit of measurement
☐	☒	A statement on whether measurements were taken from distinct samples or whether the same sample was measured repeatedly
☐	☒	The statistical test(s) used AND whether they are one- or two-sided<br>*Only common tests should be described solely by name; describe more complex techniques in the Methods section.*
☒	☐	A description of all covariates tested
☒	☐	A description of any assumptions or corrections, such as tests of normality and adjustment for multiple comparisons
☐	☒	A full description of the statistical parameters including central tendency (e.g. means) or other basic estimates (e.g. regression coefficient) AND variation (e.g. standard deviation) or associated estimates of uncertainty (e.g. confidence intervals)
☐	☒	For null hypothesis testing, the test statistic (e.g. F, t, r) with confidence intervals, effect sizes, degrees of freedom and P value noted<br>*Give P values as exact values whenever suitable.*
☒	☐	For Bayesian analysis, information on the choice of priors and Markov chain Monte Carlo settings
☒	☐	For hierarchical and complex designs, identification of the appropriate level for tests and full reporting of outcomes
☒	☐	Estimates of effect sizes (e.g. Cohen's d, Pearson's r), indicating how they were calculated

Our web collection on statistics for biologists contains articles on many of the points above.

Software and code

Policy information about availability of computer code

Data collection	Bioluminescent imaging data were obtained by using the IVIS 200 Imaging System (Perkin Elmer). Micro-computed tomography-based bone scanning data were collected by using the Bruker micro-CT SkyScan 1276 system. Quantitative PCR data were obtained by using the Bio-Rad CFX96 Real-Time System. H&E, IHC, and TRAP staining images were scanned by using the Leica Biosystems APERIO CS2. Luciferase activity data were collected by using the Biotek Synergy 2 Microplate Reader. Fluorescence images were obtained by using Zeiss LSM880 confocal microscope.
Data analysis	Bioluminescent imaging data were analyzed by using Living Image software (Version 4.7, Perkin Elmer). Fluorescence images were processed with Zen 2.6 (Zeiss) software. Backward projection datasets of femurs by µCT scanning were reconstructed by using Insta-Recon software (Bruker microCT, Kontich, Belgium). Bone mineral density (BMD), trabecular bone volume per tissue volume (BV/TV), trabecular number (Tb.N), and trabecular thickness (Tb.th) were determined by using CTAn software (Version 1.18 8.0+, Bruker microCT, Belgium). 3D models of µCT data were created by using CTVox software (Version 3.3.0, Bruker microCT, Belgium). Quantitative analysis of osteolytic lesions was performed by using ImageJ (Version 1.53m). Bone histomorphometry and MAR were analyzed by using Bioquant OSTEO II software (Bioquant Nashville). Statistical analysis was performed by using Graphpad Prism (Version 9.0.0). Bioinformatic analysis was performed by using R language (Version 4.3.2).

For manuscripts utilizing custom algorithms or software that are central to the research but not yet described in published literature, software must be made available to editors and reviewers. We strongly encourage code deposition in a community repository (e.g. GitHub). See the Nature Portfolio guidelines for submitting code & software for further information.

Data

Policy information about availability of data

All manuscripts must include a data availability statement. This statement should provide the following information, where applicable:

- Accession codes, unique identifiers, or web links for publicly available datasets
- A description of any restrictions on data availability
- For clinical datasets or third party data, please ensure that the statement adheres to our policy

All data supporting the findings of this study are available within the paper and the Source Data file. Source data are provided with this paper. Previously published data used in this study are E-MTAB-3819: https://www.ebi.ac.uk/biostudies/arrayexpress/studies/E-MTAB-3819; E-MTAB-7391: https://www.ebi.ac.uk/biostudies/arrayexpress/studies/E-MTAB-7391; GSE38747: https://www.ncbi.nlm.nih.gov/geo/query/acc.cgi?acc=GSE38747; GSE190772: https://www.ncbi.nlm.nih.gov/geo/query/acc.cgi?acc=GSE190772; GSE162454: https://www.ncbi.nlm.nih.gov/geo/query/acc.cgi?acc=GSE162454; and GSE169396: https://www.ncbi.nlm.nih.gov/geo/query/acc.cgi?acc=GSE169396.

Research involving human participants, their data, or biological material

Policy information about studies with human participants or human data. See also policy information about sex, gender (identity/presentation), and sexual orientation and race, ethnicity and racism.

Reporting on sex and gender	N/A
Reporting on race, ethnicity, or other socially relevant groupings	N/A
Population characteristics	N/A
Recruitment	N/A
Ethics oversight	N/A

Note that full information on the approval of the study protocol must also be provided in the manuscript.

Field-specific reporting

Please select the one below that is the best fit for your research. If you are not sure, read the appropriate sections before making your selection.

[x] Life sciences [ ] Behavioural & social sciences [ ] Ecological, evolutionary & environmental sciences

For a reference copy of the document with all sections, see nature.com/documents/nr-reporting-summary-flat.pdf

Life sciences study design

All studies must disclose on these points even when the disclosure is negative.

Sample size	No sample size calculation was performed. For in vivo and in vitro studies, sample sizes were determined based on our previous publications and preliminary experiments. Sample sizes were chosen based on empirical values that were sufficient to detect meaningful biological differences with good reproducibility.
Data exclusions	No data or animals were excluded from data analysis.
Replication	For animal experiments, multiple independent repeats (individual mice) were included. For other experiments, biological repeats were used and each experiment was repeated at least three times with similar results.
Randomization	Randomization was not performed due to the pre-treatment of cells or inherent differences in mouse genotypes. Age- and sex-matched mice of different genotypes were used for animal experiments (all animal experiments used genetically engineered mouse models).
Blinding	For cell-based experiments, blinding was not performed, because the investigator needed to know the groups to load the samples or perform the assay. Blinding was performed in some of the animal experiments: for the analysis of bone parameters, the technician in the core facility performed the procedure and analysis without knowing the specific groups. For bone metastasis assays and the LPS-induced inflammatory osteoporosis model, blinding was not performed, because the investigator needed to know the groups in order to perform the study.

Reporting for specific materials, systems and methods

We require information from authors about some types of materials, experimental systems and methods used in many studies. Here, indicate whether each material, system or method listed is relevant to your study. If you are not sure if a list item applies to your research, read the appropriate section before selecting a response.

Materials & experimental systems

n/a	Involved in the study
☐	☒ Antibodies
☐	☒ Eukaryotic cell lines
☒	☐ Palaeontology and archaeology
☐	☒ Animals and other organisms
☒	☐ Clinical data
☒	☐ Dual use research of concern
☒	☐ Plants

Methods

n/a	Involved in the study
☒	☐ ChIP-seq
☒	☐ Flow cytometry
☒	☐ MRI-based neuroimaging

Antibodies

Antibodies used

Primary antibodies used for immunoblotting:
Nfatc1 (1:1000, Santa Cruz Biotechnology, sc-7294, RRID: AB_2152503),
Tead1 (1:1000, Cell Signaling Technology, 12292S, RRID: AB_2797873),
Tead2 (1:1000, Proteintech, 21159-1-AP, RRID: AB_2861186),
Tead3 (1:1000, Proteintech, 13120-1-AP, RRID: AB_2203068),
Tead4 (1:1000, Proteintech, 12418-1-AP, RRID: AB_2203074),
Ctsk (1:1000, Proteintech, 11239-1-AP, RRID: AB_2245581),
Mitf (1:1000, Cell Signaling Technology, 12590S, RRID: AB_2616024),
Yap (1:1000, Cell Signaling Technology, 12395S, RRID: AB_2797897),
c-Fos (1:1000, Proteintech, 66590-1-Ig, RRID: AB_2881950),
c-Jun (1:1000, Cell Signaling Technology, 9165S, RRID: AB_2130165 ; 1:1000, Proteintech, 24909-1-AP, RRID: AB_2860574),
phospho-c-Jun (S63) (1:1000, Cell Signaling Technology, 91952S, RRID: AB_2893112),
p65 (1:1000, Cell Signaling Technology, 8242S, RRID: AB_10859369),
phospho-p65 (S536) (1:1000, Cell Signaling Technology, 3033S, RRID: AB_331284).
Erk1/2 (1:1000, Cell Signaling Technology, 4695S, RRID: AB_390779),
phospho-Erk1/2 (Thr202/Tyr204) (1:1000, Cell Signaling Technology, 4370S, RRID: AB_2315112),
Jnk (1:1000, Cell Signaling Technology, 9252S, RRID: AB_2250373),
phospho-Jnk (Thr183/Tyr185) (1:1000, Cell Signaling Technology, 4668S, RRID: AB_823588),
IκBα (1:1000, Cell Signaling Technology, 9242S, RRID: AB_331623),
Creb1 (1:1000, Cell Signaling Technology, 9197S, RRID: AB_331277; 1:1000, Proteintech, 12208-1-AP, RRID: AB_2245417),
p38 (1:1000, Cell Signaling Technology, 8690S, RRID: AB_10999090),
Cas9 (1:1000, BioLegend, 844301, RRID: AB_2749904),
Enterobacterio Phage MS2 Coat Protein (MCP) (1:1000, Sigma, ABE76-I, RRID: AB_2827507),
FLAG tag (1:10000, Sigma, F7425, RRID: AB_439687; 1:10000, Sigma, F3165, RRID: AB_259529),
MYC tag (1:2000, Cell Signaling Technology, 2278, RRID: AB_490778; 1:2000, Santa Cruz Biotechnology, sc-40, RRID: AB_627268),
HA tag (1:2000, Cell Signaling Technology, 3724S, RRID: AB_1549585; 1:5000, Santa Cruz Biotechnology, sc-7392, RRID: AB_627809),
Hsp90 (1:2000, BD Biosciences, 610419, RRID: AB_397799),
β-tubulin (1:2000, Proteintech, 10068-1-AP, RRID: AB_2303998),
β-actin (1:4000, Santa Cruz Biotechnology, sc-47778, RRID: AB_626632),
Gapdh (1:4000, Santa Cruz Biotechnology, sc-365062, RRID: AB_10847862),
Lamin B1 (1:1000, Cell Signaling Technology, 13435S, RRID: AB_2737428).

Secondary antibodies used for immunoblotting:
Anti-mouse IgG, horseradish peroxidase linked whole antibody (from sheep) (Cytia, NXA931, RRID: AB_772209),
Anti-rabbit IgG, horseradish peroxidase linked whole antibody (from donkey) (Cytia, NA934, RRID: AB_772206).

Antibodies used for IHC:
RFP (1:400, Abcam, ab62341, RRID: AB_945213)

Antibodies used for pulldown of proteins:
Nfatc1 (1:100, Invitrogen, MA3024, RRID: AB_2236037)
Tead3 (1:50, Proteintech, 13120-1-AP, RRID: AB_2203068)
pan-Tead (1:50, Cell Signaling Technology, 13295S, RRID: AB_2687902)
c-Myc Agarose Affinity Gel antibody produced in rabbit (40uL/per sample, Sigma, A7470, RRID: AB_10109522)

Validation

All antibodies used are commercially available and validated by the manufacturers. Pre-validated antibodies were purchased from reputable sources. All proteins are well studied and all antibodies are widely used in the literature. The experiments included appropriate controls. We validated the TEAD3-specific antibody by using two independent TEAD3 shRNAs and two siRNAs.

Primary antibodies used for immunoblotting:
Nfatc1 (1:1000, Santa Cruz Biotechnology, sc-7294, RRID: AB_2152503), https://www.scbt.com/p/nfatc1-antibody-7a6
Tead1 (1:1000, Cell Signaling Technology, 12292S, RRID: AB_2797873), https://www.cellsignal.com/products/primary-antibodies/tead1-d9x2l-rabbit-mab/12292

Tead2 (1:1000, Proteintech, 21159-1-AP, RRID: AB_2861186), https://www.ptglab.com/products/TEAD2-Antibody-21159-1-AP.htm
Tead3 (1:1000, Proteintech, 13120-1-AP, RRID: AB_2203068), https://www.ptglab.com/products/TEAD3-Antibody-13120-1-AP.htm
Tead4 (1:1000, Proteintech, 12418-1-AP, RRID: AB_2203074), https://www.ptglab.com/products/TEAD4-Antibody-12418-1-AP.htm
Ctsk (1:1000, Proteintech, 11239-1-AP, RRID: AB_2245581), https://www.ptglab.com/products/CTSK-Antibody-11239-1-AP.htm
Mitf (1:1000, Cell Signaling Technology, 12590S, RRID: AB_2616024), https://www.cellsignal.com/products/primary-antibodies/mitf-d5g7v-rabbit-mab/12590
Yap (1:1000, Cell Signaling Technology, 12395S, RRID: AB_2797897), https://www.cellsignal.com/products/primary-antibodies/yap-1a12-mouse-mab/12395
c-Fos (1:1000, Proteintech, 66590-1-Ig, RRID: AB_2881950), https://www.ptgcn.com/products/FOS-Antibody-66590-1-Ig.htm
c-Jun (1:1000, Cell Signaling Technology, 9165S, RRID: AB_2130165), https://www.cellsignal.com/products/primary-antibodies/c-jun-60a8-rabbit-mab/9165
c-Jun (1:1000, Proteintech, 24909-1-AP, RRID: AB_2860574), https://www.ptgcn.com/products/JUN-Antibody-24909-1-AP.htm
phospho-c-Jun (S63) (1:1000, Cell Signaling Technology, 91952S, RRID: AB_2893112), https://www.cellsignal.com/products/primary-antibodies/phospho-c-jun-ser63-e6i7p-xp-rabbit-mab/91952
p65 (1:1000, Cell Signaling Technology, 8242S, RRID: AB_10859369), https://www.cellsignal.com/products/primary-antibodies/nf-kb-p65-d14e12-xp-rabbit-mab/8242
phospho-p65 (S536) (1:1000, Cell Signaling Technology, 3033S, RRID: AB_331284), https://www.cellsignal.com/products/primary-antibodies/phospho-nf-kb-p65-ser536-93h1-rabbit-mab/3033
Erk1/2 (1:1000, Cell Signaling Technology, 4695S, RRID: AB_390779), https://www.cellsignal.com/products/primary-antibodies/p44-42-mapk-erk1-2-137f5-rabbit-mab/4695
phospho-Erk1/2 (Thr202/Tyr204) (1:1000, Cell Signaling Technology, 4370S, RRID: AB_2315112), https://www.cellsignal.com/products/primary-antibodies/phospho-p44-42-mapk-erk1-2-thr202-tyr204-d13-14-4e-xp-rabbit-mab/4370
Jnk (1:1000, Cell Signaling Technology, 9252S, RRID: AB_2250373), https://www.cellsignal.com/products/primary-antibodies/sapk-jnk-antibody/9252
phospho-Jnk (Thr183/Tyr185) (1:1000, Cell Signaling Technology, 4668S, RRID: AB_823588), https://www.cellsignal.com/products/primary-antibodies/phospho-sapk-jnk-thr183-tyr185-81e11-rabbit-mab/4668
IκBα (1:1000, Cell Signaling Technology, 9242S, RRID: AB_331623), https://www.cellsignal.com/products/primary-antibodies/ikba-antibody/9242
Creb (1:1000, Cell Signaling Technology, 9197S, RRID: AB_331277), https://www.cellsignal.com/products/primary-antibodies/creb-48h2-rabbit-mab/9197
Creb (1:1000, Proteintech, 12208-1-AP, RRID: AB_2245417), https://www.ptgcn.com/products/CREB1-Antibody-12208-1-AP.htm
p38 (1:1000, Cell Signaling Technology, 8690S, RRID: AB_10999090), https://www.cellsignal.com/products/primary-antibodies/p38-mapk-d13e1-xp-rabbit-mab/8690
Cas9 (1:1000, BioLegend, 844301, RRID: AB_2749904), https://www.biolegend.com/en-ie/products/purified-anti-crispr-cas9-antibody-11774?GroupID=BLG15641
Enterobacterio Phage MS2 Coat Protein (MCP) (1:1000, Sigma, ABE76-I, RRID: AB_2827507), https://www.sigmaaldrich.com/US/en/product/mm/abe76im
FLAG tag (1:10000, Sigma, F7425, AB_439687; 1:10000, Sigma, F3165, RRID: AB_259529), https://www.sigmaaldrich.com/US/en/product/sigma/f3165
MYC tag (1:2000, Cell Signaling Technology, 2278, RRID: AB_490778), https://www.cellsignal.com/products/primary-antibodies/myc-tag-71d10-rabbit-mab/2278
MYC tag (1:2000, Santa Cruz Biotechnology, sc-40, RRID: AB_627268), https://www.scbt.com/p/c-myc-antibody-9e10
HA tag (1:2000, Cell Signaling Technology, 3724S, RRID: AB_1549585), https://www.cellsignal.com/products/primary-antibodies/ha-tag-c29f4-rabbit-mab/3724
HA tag (1:5000, Santa Cruz Biotechnology, sc-7392, RRID: AB_627809), https://www.scbt.com/p/ha-probe-antibody-f-7
Hsp90 (1:2000, BD Biosciences, 610419, RRID: AB_397799), https://www.bdbiosciences.com/en-ca/products/reagents/microscopy-imaging-reagents/immunofluorescence-reagents/purified-mouse-anti-hsp90.610419
β-tubulin (1:2000, Proteintech, 10068-1-AP, RRID: AB_2303998), https://www.ptgcn.com/products/TUBB3-Antibody-10068-1-AP.htm
β-actin (1:4000, Santa Cruz Biotechnology, sc-47778, RRID: AB_626632), https://www.scbt.com/p/beta-actin-antibody-c4
Gapdh (1:4000, Santa Cruz Biotechnology, sc-365062, RRID: AB_10847862), https://www.scbt.com/p/gapdh-antibody-g-9
Lamin B1 (1:1000, Cell Signaling Technology, 13435S, RRID: AB_2737428), https://www.cellsignal.com/products/primary-antibodies/lamin-b1-d9v6h-rabbit-mab/13435

Secondary antibodies used for immunoblotting:
Anti-mouse IgG, horseradish peroxidase linked whole antibody (from sheep) (Cytia, NXA931, RRID: AB_772209), https://www.cytivalifesciences.com/en/us/shop/protein-analysis/blotting-and-detection/blotting-standards-and-reagents/amersham-ecl-hrp-conjugated-antibodies-p-06260
Anti-rabbit IgG, horseradish peroxidase linked whole antibody (from donkey) (Cytia, NA934, RRID: AB_772206), https://www.cytivalifesciences.com/en/us/shop/protein-analysis/blotting-and-detection/blotting-standards-and-reagents/amersham-ecl-hrp-conjugated-antibodies-p-06260

Antibodies used for IHC:
RFP (1:400, Abcam, ab62341, RRID: AB_945213), https://www.abcam.com/products/primary-antibodies/rfp-antibody-ab62341.html

Antibodies used for pulldown of proteins:
Nfatc1 (1:100, Invitrogen, MA3024, RRID: AB_2236037), https://www.thermofisher.com/antibody/product/NFATC1-Antibody-clone-7A6-Monoclonal/MA3-024
Tead3 (1:50, Proteintech, 13120-1-AP, RRID: AB_2203068), https://www.ptglab.com/products/TEAD3-Antibody-13120-1-AP.htm.
pan-Tead (1:50, Cell Signaling Technology, 13295S, RRID: AB_2687902), https://www.cellsignal.com/products/primary-antibodies/pan-tead-d3f7l-rabbit-mab/13295

c-Myc Agarose Affinity Gel antibody produced in rabbit (40uL/per sample, Sigma, A7470, RRID: AB_10109522): https://www.sigmaaldrich.com/US/en/product/sigma/a7470

Eukaryotic cell lines

Policy information about cell lines and Sex and Gender in Research

Cell line source(s)	The HEK293T (female) cell line was from Li Ma's lab stock (originally from the American Type Culture Collection, ATCC, CRL-3216). The L929 (male) cell line was from Dr. Dihua Yu (MD Anderson Cancer Center, Houston, TX). The RAW264.7 (male) and EO771 (female) cell lines were from Dr. Liuqing Yang (MD Anderson Cancer Center, Houston, TX). The U937 (male) cell line was from Dr. Xiang Zhang (Baylor College of Medicine, Houston, TX). The B16F1 (male) cell line was from MD Anderson's Cytogenetics and Cell Authentication Core.
Authentication	Short tandem repeat (STR) profiling was done by ATCC and MD Anderson's Cytogenetics and Cell Authentication Core.
Mycoplasma contamination	All cell lines were confirmed to be mycoplasma free with a mycoplasma detection kit and treated with Plasmocin for the prevention of mycoplasma contamination.
Commonly misidentified lines (See ICLAC register)	None.

Animals and other research organisms

Policy information about studies involving animals; ARRIVE guidelines recommended for reporting animal research, and Sex and Gender in Research

Laboratory animals	Species: mouse<br><br>Gender: male and female<br><br>Strains:<br>Malat1+/+ mice (C57BL/6)<br>Malat1-/- mice (C57BL/6)<br>Malat1Tg/Tg mice (C57BL/6)<br>Malat1-/-;Malat1Tg/Tg mice (C57BL/6)<br><br>Ages and procedures:<br>(1) Intratibial injection: 6-month-old male mice for B16F1 cells, 3-month-old female for EO771 cells<br>(2) LPS-induced inflammatory osteoporosis model: 8-week-old female mice<br>(3) µCT analysis of bone parameters: 6-month-old male and female mice; 3-month-old female mice<br><br>Animals were housed at 70 °F-74 °F (set point: 72 °F) with 40%-55% humidity (set point: 45%). The light cycle of animal rooms is 12 hours of light and 12 hours of dark.
Wild animals	No wild animals were used.
Reporting on sex	Both male and female mice were used for the measurements of bone density and other bone parameters. Male mice were used as recipients of B16F1 cells and female mice were used as recipients of EO771 cells.
Field-collected samples	No field-collected samples were used.
Ethics oversight	All animal studies were performed in accordance with a protocol (PI: Li Ma) approved by the Institutional Animal Care and Use Committee of MD Anderson Cancer Center.

Note that full information on the approval of the study protocol must also be provided in the manuscript.

Plants

Seed stocks	N/A
Novel plant genotypes	N/A
Authentication	N/A
